# Toward a clear relationship between mechanical signals and bone adaptation

**DOI:** 10.1016/j.mbm.2025.100115

**Published:** 2025-02-01

**Authors:** Chenlu Wang, Ruisen Fu, Haisheng Yang

**Affiliations:** Department of Biomedical Engineering, College of Chemistry and Life Science, Beijing University of Technology, Beijing, 100124, China

**Keywords:** Bone adaptation, *In vivo* loading, Animal models, Mechanical signals, Osteocyte

## Abstract

Bone adapts according to the mechanical environment, and this adaptation can be visualized by altering its shape, size, and microarchitecture. Bone adaptation was recognized more than a century ago, with a description presented in *The Law of Bone Remodeling*. Furthermore, the conceptual model of “*The Mechanostat*” provides a quantitative relationship between the magnitude of bone tissue deformation (strain) and bone adaptive responses. However, upon maintaining a constant strain magnitude, various bone responses were observed experimentally under different loading parameters (e.g., frequency, rate, number of load cycles, rest insertion, and waveform). Nevertheless, the precise relationship between mechanical signals and bone adaptation remains unclear. Accordingly, we reviewed *in vivo* loading studies to determine the quantitative relationships between various mechanical signals and bone adaptive responses in various animal loading models. Additionally, we explored how these relationships are influenced by pathophysiological factors, such as age, sex, and estrogen deficiency. Moreover, mechanistic studies that consider cellular mechanical microenvironments to explain these quantitative relationships are discussed. A general formula that considers the bone adaptive response as a function of different loading parameters was proposed. This review may enhance our understanding of bone adaptation and offer guidance for clinicians to develop effective mechanotherapies to prevent bone loss.

## Graphical abstract

**Fig.** Overview of the present review paper. In this review, we first started with a summary of typical animal models used for *in vivo* loading, and then comprehensively summarized the results regarding the relationships between different loading parameters and bone responses, which have been identified by those *in vivo* animal loading experiments. Next, we examined how these relationships were influenced by pathophysiological factors such as age, sex, estrogen deficiency, disuse, and different disease states. Lastly, we discussed underlying mechanisms governing these load-bone adaptation relationships.

**Figure:**
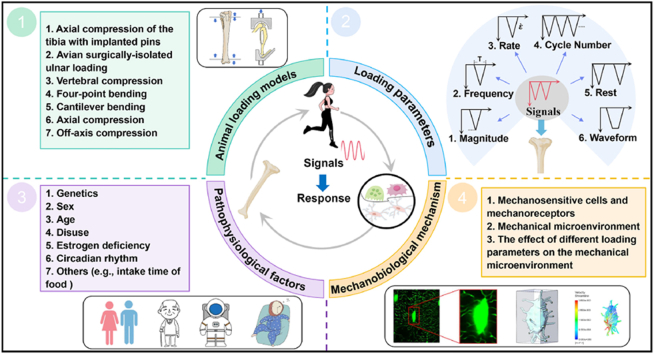

## 1 Introduction

**Fig. 1:**
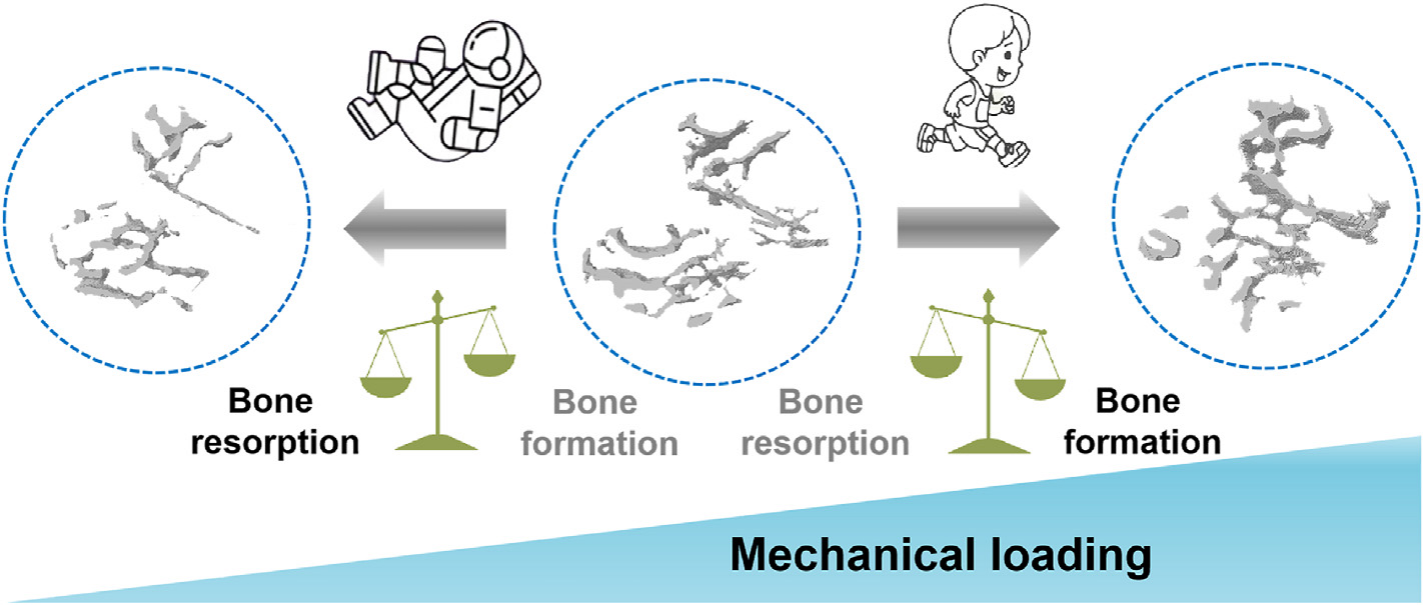
Bone formation and resorption are regulated by mechanical loading. A reduction in mechanical loading (e.g., spaceflight) leads to bone loss, while an increase in mechanical loading (e.g., exercise) could increase bone mass.

Bone supports the skeletal system and facilitates multiple functions, including locomotion, organ protection, appropriate mineral storage, and maintenance of hematopoietic cells.^1^ Bones reshape their appearance and maintain structural and functional integrity through two basic physiological activities: bone modeling and bone remodeling.^2^ Bone modeling refers to the process of shaping skeletal elements and ensuring the acquisition of appropriate bone morphology and mass.^2^, ^3^, ^4^ During this process, bone resorption and formation, governed by osteoclasts and osteoblasts, respectively, occur in an uncoupled manner on separate surfaces.^5^ Bone remodeling ensures tissue turnover while maintaining bone mass; it also allows adaptation to mechanical loading and requirements of calcium and phosphate metabolism in the mature skeleton.^3^^,^^4^^,^^6^ Bone resorption and formation are tightly coupled.^7^ A reduction in mechanical stimuli (e.g., space flight^8^ and long-term bed rest^9^) results in an imbalance between bone formation and resorption, eventually leading to bone loss or osteoporosis^10^ (Fig. 1). In contrast, enhanced mechanical loading (e.g., exercise) could promote bone formation, thereby increasing bone mass.^11^^,^^12^

**Fig. 2:**
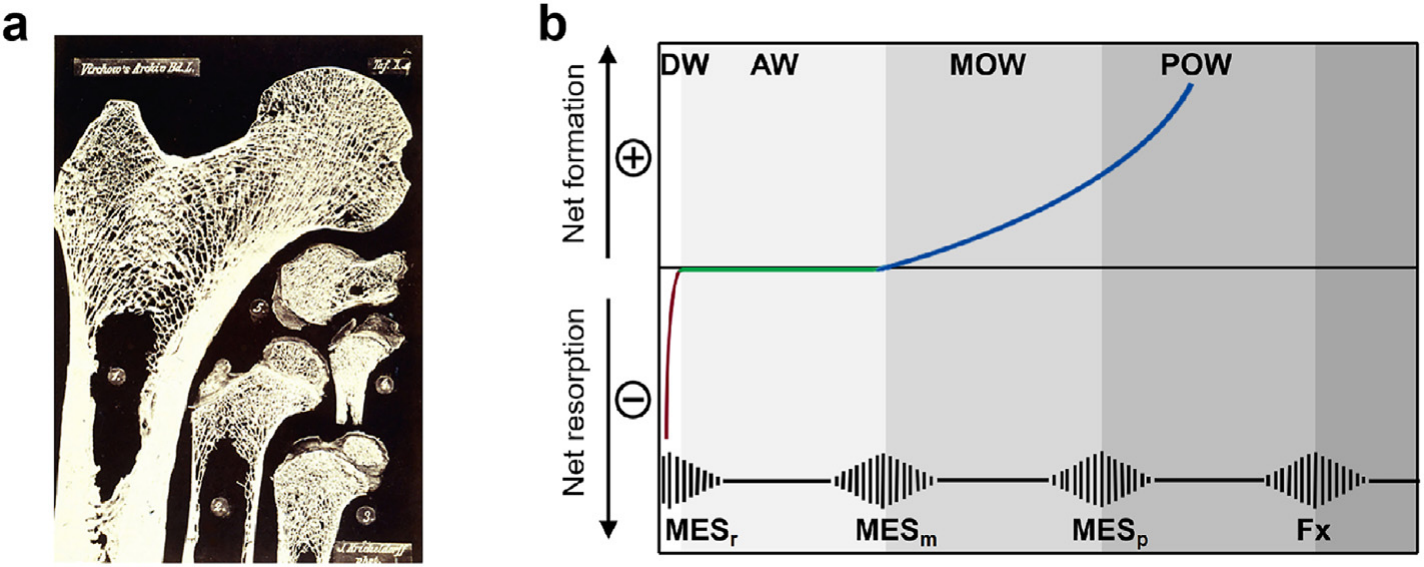
Mechanical adaptation of bone. (**a**) Julius Wolff popularized the idea that trabecular bone is aligned along the lines of stress (adapted from ^22^). (**b**) Illustration of the “*mechanostat*” hypothesis (modified from ^24^). The horizontal axis depicts peak bone strain, and the vertical axis depicts net loss (−) or gain (+) of bone mass. The lower pulsed line shows the threshold values of “minimum effective strain” (MES) for remodeling (MESr), modeling (MESm), microdamage (MESp), and fracture strain (Fx). The regions labeled at the top represent the disuse window (DW), adapted window (AW), mild overload window (MOW), and pathologic overload window (POW).

In the 15th century, Galileo discovered that the shape and size of bones are related to mechanical forces.^13^^,^^14^ Von Meyer found arched trabecular patterns in sagittally sectioned human first metatarsal and calcaneus, and Culmann suggested that the patterns appeared to be aligned along the directions of principal stress produced by functional loading.^15^ Inspired by von Meyer et al., Julius Wolff hypothesized that “the direction and pattern of loading influences and/or controls the pattern of the cancellous framework”^16^ (Fig. 2a). Subsequently, Roux recognized that functional adaptation of the bone is a dynamic and self-regulating process.^17^, ^18^, ^19^ Thomson and Frost also proposed that loading-induced tissue deformation (strain) affects bone cells, thus regulating the adaptive response of the bone.^20^ In summary, Roesler highlighted three key concepts regarding the ability of bone to adapt to changing mechanical loads: (1) bone structure optimizes strength with respect to the amount of material used, (2) trabeculae line up with principal stress directions, and (3) these are accomplished by a self-regulating system of bone cells responding to a mechanical stimulus.^17^^,^^21^ Currently, Wolff's law remains a rather poorly defined “law,” but it roughly includes these three core principles.^21^^,^^22^ Furthermore, Frost proposed a conceptual model, “*The Mechanostat*,” which quantitatively described the relationship between strain magnitude and bone adaptative responses.^23^^,^^24^ “The Mechanostat” theory indicates that bone mass and architecture are locally controlled by bone cells, corresponding to the difference between the actual mechanical strain and different thresholds (set points). Accordingly, the bone formed when actual strain exceeded the set point and was resorbed when the actual strain was below the set point; in both cases, the strains were restored to the level of the set point (Fig. 2b). Frost also noted that certain bone diseases, such as osteogenic imperfecta, may have higher set points.^25^ Additionally, set points may be similar across species.^26^^,^^27^ “The Mechanostat” theory substantially advances the understanding of bone mechanical adaptation. Currey also recognized that a change in stiffness affects the strain induced by the same mechanical forces.^28^^,^^29^

The development of *in vivo* strain gauging technology,^30^^,^^31^ which allows the measurement of local strain on the bones of living animals/human beings, and the utilization of functional disuse models,^32^, ^33^, ^34^ has helped researchers to better understand the relationship between strain and bone adaptation. For example, Lanyon et al. implanted electrical-resistance strain gauges on the tibial bone surfaces of humans and mice to quantify the strain stimuli generated during various activities.^35^^,^^36^ Turner et al. found that osteogenic responses were enhanced with increases in strain magnitude.^37^ Rubin and Lanyon reported that new bone formation occurred at peak strain sites.^26^

**Fig. 3:**
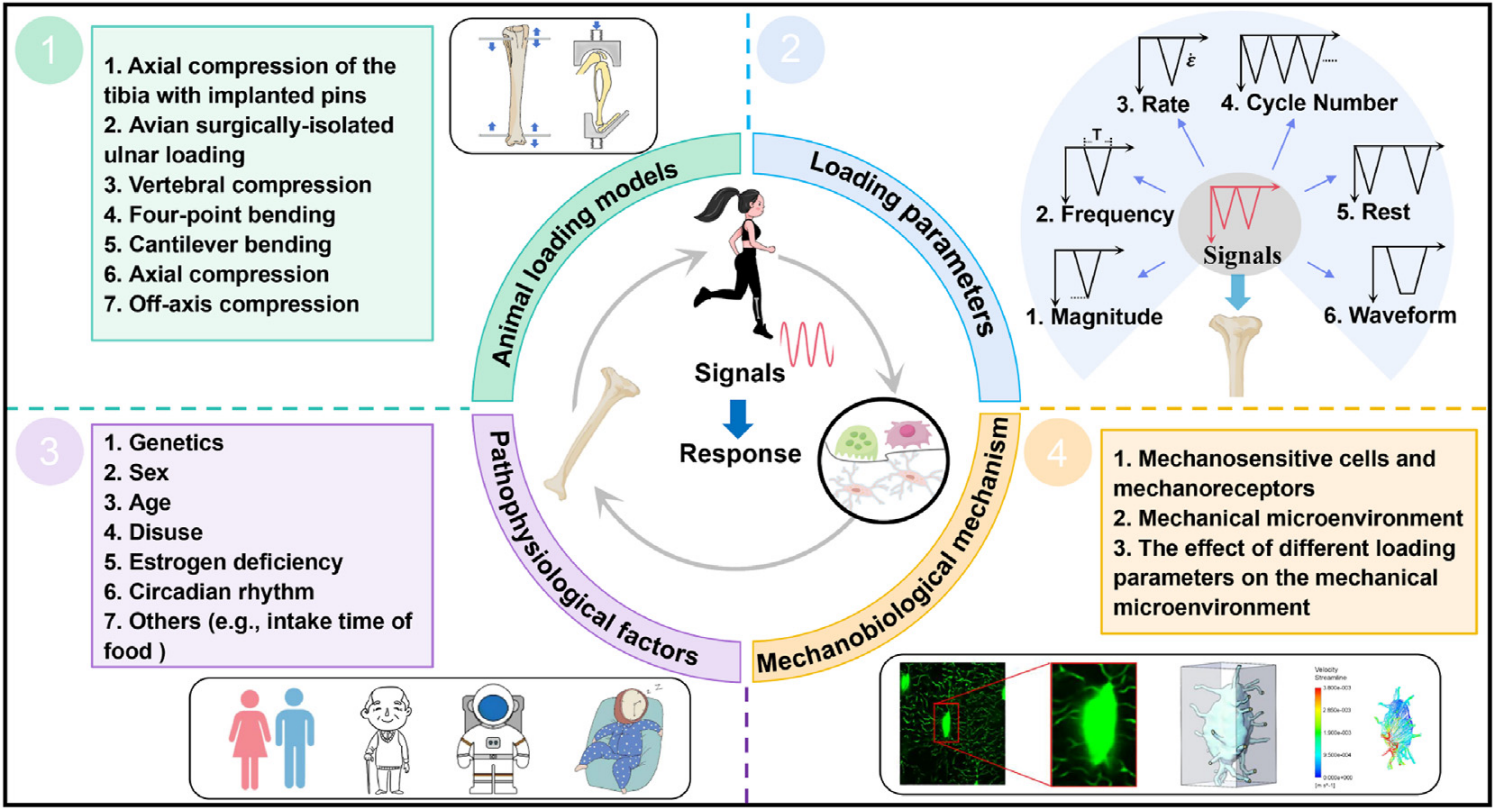
Overview of the present review paper. This review summarizes typical animal models used for *in vivo* mechanical loading, highlighting the relationships between loading parameters and bone responses. Subsequently, the review examines how these relationships are affected by factors such as age, sex, estrogen deficiency, disuse, and disease states and concludes with a discussion on the underlying mechanisms that govern load-induced bone adaptation.

Undoubtedly, load/strain magnitude is an important factor governing bone mechanoadaptation. However, the load must be dynamic to induce a bone response.^38^^,^^39^ Likewise, several *in vivo* loading experiments^40^^,^^41^ have indicated that bone responses to externally applied mechanical loading are influenced by numerous loading parameters other than strain magnitude, such as frequency, rate, number of load cycles, rest insertion, and waveform. Although emerging animal loading studies have enhanced our understanding of bone adaptation to mechanical loading, the relationship between different mechanical signals and adaptive bone responses remains unclear. In this review, we first summarize the typical animal models used for *in vivo* loading (Fig. 3) in Section 2. In Section 3, we comprehensively summarize the results regarding the relationships between different loading parameters and bone responses, which were identified in the *in vivo* animal loading experiments (Fig. 3). In Section 4, we examine how these relationships are affected by pathophysiological factors, such as genetics, sex, age, disuse, estrogen deficiency, nutrition, and circadian rhythm (Fig. 3). Finally, in Section 5, we discuss the cellular biomechanical mechanisms that govern the relationship between various mechanical signals and bone adaptation (Fig. 3). This review could enhance our understanding of bone mechanoadaptation and ultimately assist orthopedic researchers and medical practitioners in designing effective biomechanical interventions, such as loading/exercise regimes, to prevent bone loss induced by aging, estrogen deficiency, or disuse.

## 2 Typical animal loading models used to examine bone responses to various loading parameters

Various *in vivo* animal loading models have been developed over the years and used to examine the influence of mechanical loading on osteogenesis. Typically, there are two types of *in vivo* loading models: intrinsic (e.g., running,^42^^,^^43^ jumping,^44^ and swimming^45^) and extrinsic, which can be further categorized into invasive and noninvasive.

Intrinsic loading models typically induce mechanical stimuli on the bones via ground reactions and muscle-related forces through various physical activities. In these experiments, animals are trained to perform a certain type of exercise to induce strains higher than those induced by daily activities.^46^ However, even within the same experimental group, the peak strain and strain distribution within the same bone under the same type of exercise regimen vary greatly among animals,^47^^,^^48^ complicating the identification of any precise relationships between mechanical signals and bone responses with this type of model.

**Fig. 4:**
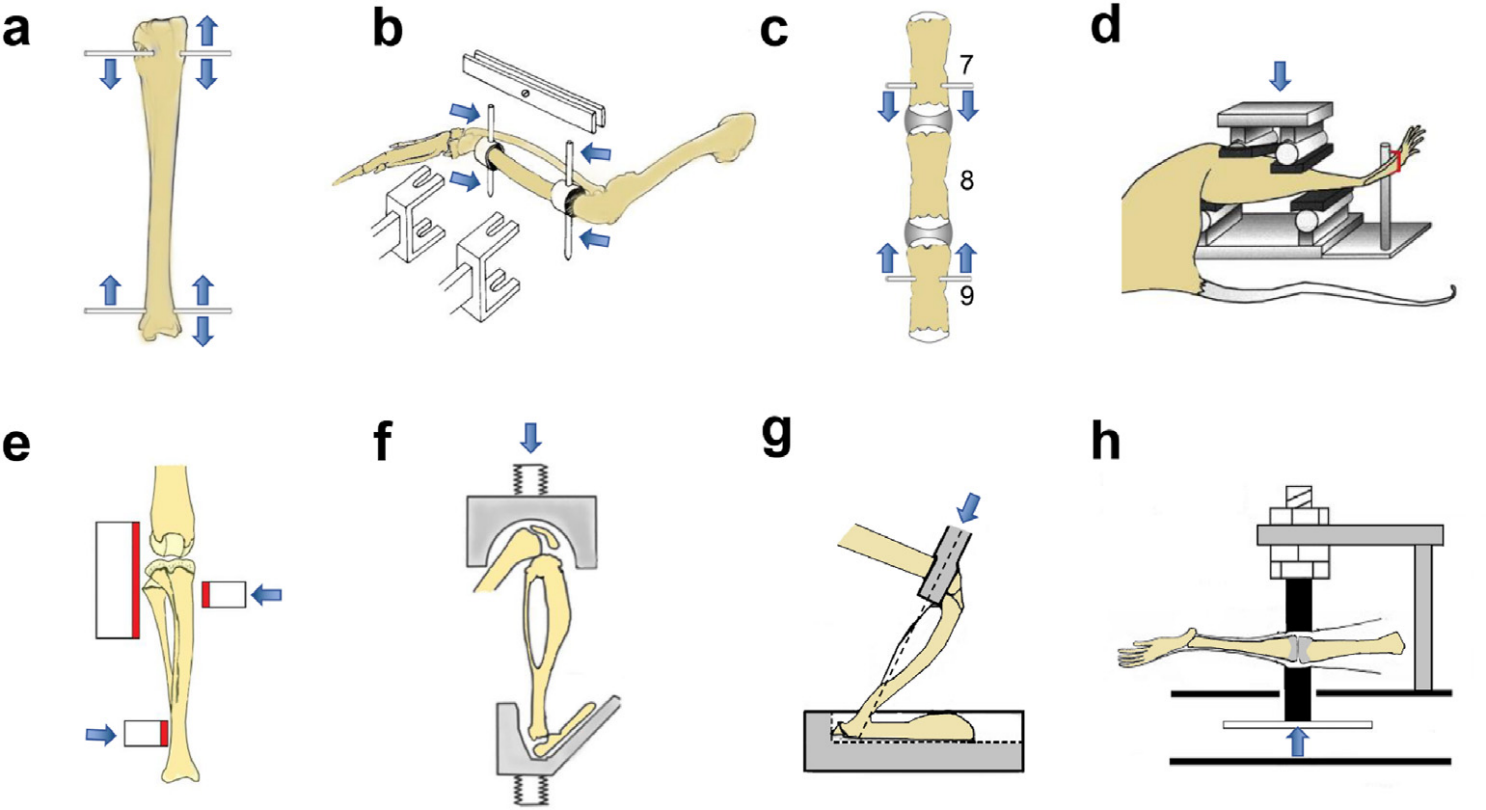
Animal loading models used to examine bone adaptation under various loading parameters (modified from ^22^). (**a**) Axial compression of the tibia with implanted pins. (**b**) Avian surgically-isolated ulnar loading. (**c**) Vertebral compression. (**d**) Four-point bending. (**e**) Cantilever bending. (**f**) Axial compression of tibia. (**g**) Off-axis compression. (**h**) Knee-loading.

In contrast, extrinsic animal loading models (including invasive and noninvasive loading models) allow for the application of controllable precise mechanical signals, facilitating the exploration of quantitative relationships between different mechanical signals and bone adaptation (Fig. 4). Additionally, the contralateral non-loaded limb can be used as a within-animal control,^49^ thereby improving the accuracy of the identified quantitative relationships. Therefore, our discussion focuses on the extrinsic loading models (Table 1).

**Table 1:** A summary of *in vivo* animal loading models commonly used to determine the quantitative relationships between various mechanical signals and bone adaptive responses.

Loading model	Stimulated bone types	Bone tested	Animal model	Complications	Advantages	Physiological loading direction?
Axial compression of tibia with implanted pin	Cortical	Tibia	Rabbit^50^	Injury-induced bone formation; technically challenging	No attenuation of mechanical signal	×
			Sheep^51^^,^^52^			
			Dog^53^			
Avian surgically-isolated ulnar loading	Cortical	Ulna	Turkey^54^	Injury-induced bone formation; technically challenging	No attenuation of mechanical signal	×
			Chicken^55^			
Vertebral compression	Cancellous	Vertebra	Rat^56^^,^^57^	Injury-induced bone formation; technically challenging	Suitable for studying the cancellous bone	√
	Cortical		Mouse^58^, ^59^, ^60^, ^61^			
Four-point bending	Cortical	Tibia	Rat^62^	Woven bone is induced at the periosteal surface	Noninvasive	×
			Mouse^63^^,^^64^			
Three-point bending	Cortical	Tibia	Mouse^65^^,^^66^	Woven bone is induced at the periosteal surface	Noninvasive	×
		The third metatarsal				
Cantilever bending	Cortical	Tibia	Mouse^67^^,^^68^	Limited strain magnitude	Noninvasive; suitable for studying the endosteum and periosteum	×
Axial compression of ulna	Cortical	Ulna	Rat^69^	Soft tissues attenuate the mechanical signal	Noninvasive	√
			Mouse^70^			
			Rabbit^71^			
Axial compression of the tibia	Cortical	Tibia	Rat^72^	Soft tissues attenuate the mechanical signal	Noninvasive	√
	Cancellous	Fibula	Mouse^73^^,^^74^^,^^75^, ^76^, ^77^, ^78^, ^79^, ^80^			
Off-axis compression	Cortical	Tibia	Mouse^81^	Technically challenging	Noninvasive; reduces the applied loading required to induce a given peak strain	×
	Cancellous					
Knee-loading	Cortical	Tibia	Mouse^82^, ^83^, ^84^	Technically challenging	Noninvasive	×
		Femur				

### 2.1 Invasive animal loading models

Hert et al. investigated how the skeleton responded to mechanical loading by exerting a load on wires placed into a rabbit's tibia.^50^ In this model, Kirschner wires were inserted into holes drilled through the proximal and distal tibial metaphyses of anesthetized rabbits, which required approximately 30 ​d for healing (Fig. 4a). After healing, the tibia was loaded in axial compression by clamping the two wires together or in mediolateral bending by clamping the lateral wire tips together and drawing the medial wire tips. This model has also been applied to other species, including sheep^51^^,^^52^ and dogs.^53^

Subsequently, Rubin et al. designed an avian ulnar loading model in which the proximal and distal metaphyseal regions of the rooster (or turkey) ulna were sawed, creating two small incisions between the central 80 ​% of the ulna and bone end.^54^ Metal caps were affixed to the two ends of the functionally isolated ulnar shaft (Fig. 4b). Finally, Steinmann pins were inserted into the pre-drilled holes in the caps to receive the actuator-generated forces.^55^ During the non-loading sessions, the pins were clamped to prevent notable deformation of the ulnar shaft. Nevertheless, the ulna was in a state of disuse between loading sessions.

Chambers et al. established a rodent vertebral compression model, where the two vertebrae adjacent to the rat 8th caudal vertebra (i.e., CV7 and CV9) were pierced and pinned using Steinmann pins.^56^, ^57^, ^58^ After implantation, the two pins were connected using an actuator that cyclically drew the two pins together, thereby applying a dynamic compressive load to CV8 through the adjacent vertebrae and intervertebral discs (Fig. 4c). This loading model was also used to examine the adaptive response of CV6 to mechanical loading, with CV5 fixed and CV7 loaded.^59^

Among the three models discussed above, the main advantages of the axial compression of the tibia with implanted pins and avian surgically isolated ulnar loading models over the vertebral compression model are as follows: 1) they are suitable for investigating cortical bone adaptation to loading, and 2) the mechanical signals generated by the actuator are transmitted through markedly rigid materials rather than through joints and soft tissues, and therefore, they are maintained at a high degree of integrity in the bone. The vertebral compression model is more suitable for studying the adaptive response of the vertebral cancellous bone to loading,^60^^,^^61^ although the actuator-generated mechanical signals must travel through the intervertebral discs; therefore, they are potentially weakened or altered upon reaching CV8.

### 2.2 Noninvasive animal loading models

Although invasive loading models have advantages in maintaining the accuracy of the mechanical signals during transmission, unexpected bone responses due to surgical interventions are one of the main concerns, potentially confounding the results of interest.^55^ To address these concerns, noninvasive loading models have been developed and implemented in rodents.

Turner et al. created the first noninvasive animal loading model,^62^ that is, a four-point bending of the rat tibia. This model was later adapted and applied to the mouse tibia.^63^^,^^64^ In this model, the tibia is located between the upper and lower fixed pads. Compressive loading was applied through the upper contact points, that resulted in a bending moment in the central diaphysis spanning between the two upper contact points (Fig. 4d). Sham loading control with no bending of the bone was achieved by placing the upper contact points directly opposite the lower contact points. A three-point bending model is similar to a four-point bending model,^64^, ^65^, ^66^ although both models usually produce a strong woven bone response on the periosteal surface, independent of the magnitude of the applied load, limiting their applicability for investigating periosteal osteogenesis.^27^^,^^62^

To avoid periosteal woven bone-forming issues, Gross et al. developed a cantilever-bending model of the mouse tibia,^67^ in which the proximal tibia of the mouse was fixed, whereas the distal tibia was pushed medially by an actuator, inducing mediolateral bending of the tibia (Fig. 4e). However, the metaphyseal region containing cancellous bone remains largely unloaded. Therefore, the model cannot be used to examine the adaptive response of cancellous bone to loading. Additionally, the magnitude of the applied load or the strain induced by the loading within the bone is limited owing to challenges in firmly fixing the knee.^68^

Currently, axial compression of the tibia or ulna is widely used to study the mechanoadaptation of bone.^69^, ^70^, ^71^, ^73^, ^74^, ^72^ This model was initially developed by Torrance et al. and was first applied to the rat ulna.^69^ The ulna was fixed between two small metal cups. One end of the cup received the elbow, while the other end received the dorsal surface of the volar-flexed wrist (Fig. 4f). Under axial compression, the diaphysis of the loaded bone is naturally bent, and most of the axial compression is transformed into a mediolateral bending moment.^75^ Therefore, the strain distribution within the bone induced by the axial compression was consistent with that measured *in vivo* during locomotion.^76^ This model has been widely used in mice with gene manipulations (such as congenic^77^ and knockout^78^, ^79^, ^80^) to elucidate the mechanobiological mechanisms of bone adaptation.

Based on the axial compressive loading model, Srinivasan et al. developed an off-axis compression model for mouse tibia.^81^ In this model, the foot of the mouse was secured in a custom footbed, with the direction of the knee and ankle joints aligned as during free walking, with the load applied to the distal femur using a loading tine (oriented 60 °from the vertical) (Fig. 4g). During the loading process, the loading tine was not aligned with the long axis of the tibia in either the anterior-posterior or medial–lateral planes, thereby achieving off-axis compressive loading.^81^ The primary advantage of this model over the axial compressive loading model is its capacity to reduce the applied loading magnitude required to induce a given peak strain.

In addition, to determine the effects of loading on bone formation at a site distant from the loading site, a knee-loading model was developed,^82^, ^83^, ^84^ in which the lateral and medial sides of the mouse knee (including the epiphysis of the proximal tibia and distal femur) were confined between the upper and lower cups (Fig. 4h). Mechanical loads were applied to the knee through the upper contact cups, keeping the tibial diaphysis distant from the loading site. This model was primarily used to study the adaptive response of the diaphyseal bone to knee loading.

In summary, each loading model has distinct advantages and limitations, and researchers should be aware of these when performing *in vivo* loading studies or interpreting the outcomes of loading models. For example, a three- or four-point bending model may induce a woven bone response on the periosteal surface. Although the cantilever bending model avoids this response, it fails to load the cancellous bone adequately, and the magnitude of the applied load or the strain induced by loading within the bone is limited.^68^ Axial compression models can generate a more physiologically mechanical environment but require greater loads,^64^^,^^72^^,^^85^ which may increase the risk of premature growth plate closure.^86^ The off-axis compression model addresses this issue by reducing the applied load magnitude, whereas the knee-loading model uniquely facilitates the investigation of adaptive responses in bones distant from the loading site.

Moreover, it should be noted that the mechanical environments (e.g., strain distribution) generated within the bone by the cantilever bending model and the three- or four-point bending models are non-physiological. Reportedly, bone responses may be more sensitive to accidental non-physiological loading than to habitual physiological loading.^87^ However, it remains unclear how non-physiological loading-induced bone responses differ from physiological loading.^88^ Therefore, when designing *in vivo* animal loading experiments, specific objectives and appropriate models must be considered to ensure the effectiveness and reliability of the experimental outcomes.

## 3 Key mechanical loading parameters affecting bone's adaptive responses

Daily activities, including physical exercise, produce mechanical signals in load-bearing bones. Mechanical signals are forces or strains as functions of time and consist of several basic parameters such as magnitude, frequency, rate, waveform, number of load cycles, and rest insertion^89^^,^^90^ (Fig. 3). The measurement of human tibial strain or ground reaction force revealed that strain-time curves vary under different forms of locomotion.^89^^,^^91^^,^^92^ Different types of exercise produce distinct loading signals or parameters. For instance, the axial peak compressive strain was 2.9 times higher during overground running than during treadmill running.^90^ The step frequency during running was higher than that during walking.^93^ The strain rate can double or triple during vigorous activities when compared with walking at a comfortable speed.^93^^,^^94^ To design effective exercise regimens to improve bone health, it is important to identify precise relationships between loading signals (or parameters) and bone adaptation. Over the last four decades, investigations have attempted to understand the “black box” of bone mechanoadaptation by altering loading parameters (inputs) and measuring bone responses (outputs) in a variety of *in vivo* animal loading models (Table 2).

**Table 2:** A summary of relevant studies and findings regarding the effects of different loading parameters on bone adaptation.

Reference	Loading form	Animal model	Loading parameters	Key findings
**Load/strain magnitude**				
Turner et al.^27^	Four-point bending	Rat tibia	27, 33, 40, 52, or 64 ​N	The amount of new woven bone and the woven bone-forming surface are independent of the magnitude of applied strain.
Turner et al.^37^	Four-point bending	Rat tibia	30, 40, 52, or 64 ​N	Older rats have a greater mechanical threshold of bone formation than adult rats.
Turner et al.^62^	Four-point bending	Rat tibia	37 ​N	The woven bone is formed in areas with high strain, and its formation can be attributed to the bending stimulus rather than pathology.
Lee et al.^70^	Axial compression	Mouse ulna	3 or 4.3 ​N	Loading to peak strains of 2000 με stimulates lamellar periosteal bone formation but no response endosteally; loading to peak strains of 3000 με induces a mixed woven/lamellar periosteal response and lamellar endosteal bone formation.
Yang et al.^74^	Axial compression	Mouse tibia	3.5, 5.2, or 7 ​N	Cancellous bones may have a greater adaptive strain threshold than cortical bone.
De souza et al.^76^	Axial compression	Mouse tibia	Applied loads between 5 and 13 ​N	A dose-dependent response to loading magnitude can be observed in the periosteal bone.
Hsieh et al.^95^	Axial compression	Rat ulna	5, 10, 15, or 20 ​N	The strain threshold varies from 1343 με proximally to 2284 με at the midshaft to 3074 με distally.
Sugiyama et al.^96^	Axial compression	Mouse tibia	0, 2, 4, 6, 8, 10, 12, or 14 ​N	The functional adaptation of bone mass/strength related to loading is basically a linear response to the bone's physiological loading.
Berman et al.^97^	Axial compression	Mouse tibia	8.8, 10.6, or 12.4 ​N	A dose-dependent relationship exists between the magnitude of the applied load and the increase in cortical and cancellous morphological parameters.
Weatherholt et al.^98^	Axial compression	Mouse tibia	5, 7, or 9 ​N	A dose–response relationship to loading magnitude can be observed within cortical bone.
Mosley et al.^99^	Axial compression	Rat ulna	5, 7, 11, 15, or 19 ​N	During the growth process, there is a substantial linear relationship between the magnitude of the applied load and the reduction of longitudinal growth.
Brodt et al.^100^	Axial compression	Mouse tibia	8, 10, or 12 ​N	A dose–response relationship is evident, with greater bone formation observed with increasing strain magnitude.
**Load/strain frequency**				
Warden et al.^41^	Axial compression	Mouse ulna	1, 5, 10, 20, or 30 ​Hz	Cortical bone adaptation to mechanical loading increases with increasing loading frequency up to 5–10 ​Hz, plateauing thereafter (non-linear).
Rubin et al.^101^	Avian surgically-isolated ulnar loading	Turkey ulnar	1 or 20 ​Hz	The degree of growth depends on the frequency of strain application, responding in a dose manner.
Hsieh et al.^102^	Axial compression	Rat ulna	1, 5, or 10 ​Hz	Cortical bone formation increases successively with increasing loading frequency up to 10 ​Hz.
Chen et al.^103^	Axial compression	Rat ulna	5, 10, or 15 ​Hz	Bone adaptation to mechanical loading increases with increasing loading frequency.
Turner et al.^104^	Four-point bending	Rat tibia	0.05, 0.1, 0.2, 0.5, 1.0, or 2.0 ​Hz	Bone formation rate increases in a dose-dependent manner at a frequency of 0.5–2 ​Hz.
Scheuren et al.^105^	Vertebral compression	Mouse CV6	2, 5, or 10 ​Hz	There is a logarithmic relationship between loading frequency and the cancellous bone adaptation.
**Load/strain rate**				
Turner et al.^38^	Four-point bending	Rat tibia	0, 0.013, 0.026, or 0.039 s^−1^	The amount of new bone formation is directly proportional to the rate of strain in the bone tissue.
Mosley et al.^106^	Axial compression	Rat ulna	0.018, 0.030, or 0.100 s^−1^	Within the physiological range, high strain rates are associated with more osteogenic response than low strain rates.
LaMothe et al.^107^	Cantilever bending	Mouse tibia	0.004, 0.020, or 0.100 s^−1^	The higher the strain rate, the stronger the adaptive response of the periosteal surface, and the adaptability of the endosteal does not increase with the strain rate.
**Number of load cycles**				
Yang et al.^40^	Axial compression	Mouse tibia	36, 216, or 1200 cycles per day	Relatively few load cycles (e.g., 36) are sufficient to induce osteogenic responses, and the incremental increase in osteogenic reactions decreases as the number of load cycles increases.
Rubin et al.^55^	Surgically-isolated ulnar loading	Rooster ulna	4, 36, 360, or 1800 cycles per day	36 cycles/day and 1800 cycles/day can also effectively promote bone formation.
Torrance et al.^69^	Axial compression	Rat ulna	20, 1200, or 12,000 cycles per day	Increasing the number of load cycles per day from 1200 to 12,000 did not induce any increase in loading-related bone formation.
Sun et al.^108^	Axial compression	Mouse tibia	60, 300, or 1200 cycles per day	Varying the daily cycle number from 60 to 1200 exerts no effect.
Umemura et al.^109^	Jumped with electric stimulus	Rat femur	5, 10, 20, 40, or 100 jumps per day	Five jumps per day are sufficient to increase bone mass, and the continued increase in the number of jumps will reduce the return on bone mass.
**Rest insertion**				
Yang et al.^40^	Axial compression	Mouse tibia	10 ​s rest inserted at −1 ​N between every 4 load cycles	10 ​s rest time between load cycles did not improve the osteogenic response of cortical or cancellous bone tissue.
Robling et al.^75^	Four-point bending	Rat tibia	Recovery time of four loads: 0, 0.5, 1, 2, 4, or 8 ​h Rest inserted between each load cycle: 0.5, 3.5, 7, or 14 ​s	Approximately 4–8 ​h of recovery is sufficient to restore full mechanosensitivity to the cells; a longer cycle interval can improve the osteogenic response more than a shorter interval.
Robling et al.^110^	Four-point bending	Rat tibia	360 cycles are delivered in 1, 2, 4, or 6 bouts (i.e., 360 ​× ​1, 180 ​× ​2, 90 ​× ​4, or 60 ​× ​6)	360 daily load cycles applied at intervals of 60 ​× ​6 or 90 ​× ​4 represent a better osteogenic stimulus than 360 cycles applied all at once.
Srinivasan et al.^111^	Surgically-isolated ulnar loading	Turkey ulna	10 ​s (zero-load) rest inserted between each load cycle	Low-magnitude mechanical loading becomes osteogenic when rest is inserted between each load cycle.
Srinivasan et al.^112^	Cantilever bending	Mouse tibia	10 ​s (zero-load) rest inserted between each load cycle	In aged bones, inserting 10 ​s of rest between low-magnitude load cycles initiates and enhances bone formation.
**Interactions of different loading parameters**				
Liu et al.^113^	Axial compression	Rat tibia	Load magnitude: 10 ​N, 20 ​N, and 40 ​N	A high strain level can markedly induce an osteogenic response in a short time while prolonging the experiment time at a medium strain level can also substantially improve bone quality.
			Load cycles: 2 weeks and 4 weeks	
Qin et al.^114^	Avian surgically-isolated ulnar loading	Turkey ulnae	30 ​Hz, 9N, 108000 cycles	Sufficient numbers of load cycles are applied at an appropriate high frequency, while an extremely low strain level can also prevent bone loss in the disused state.
LaMothe et al.^115^	Cantilever bending	Mouse tibia	Rest insertion: 10 ​s (zero-load)	Although the addition of rest insertion reduces the number of cycles by 10 times, the combination of rest insertion and high-frequency loading enhances osteogenesis.
			Load cycles: 100 or 10 cycles per day	
Srinivasan et al.^116^	Cantilever bending	Mouse tibia	Rest insertion: 10 ​s (zero-load)	Rest intervals reduce the number of loading bouts required to enhance bone formation.
			Load bouts: Three bouts or one bout per week	
Srinivasan et al.^117^	Cantilever bending	Mouse tibia	Rest insertion: 10 ​s (zero-load)	Rest insertion loading rapidly amplifies the response of bone to small increases in strain and load cycles.
			Load magnitude: 1000, 1250, or 1600 με	
			Load cycles: 10, 50, or 250 cycles per day	

### 3.1 Load/strain magnitude

Strain magnitude is a crucial factor that affects the mechanical adaptive response of bone.^118^ Generally, the peak strain induced within the bone is proportional to the magnitude of the applied load within the linear elastic range of the bone. The load-strain magnitude relationship also depends on the animal loading model and is affected by multiple factors, such as age and sex.^64^^,^^72^^,^^85^^,^^119^^,^^120^

Therefore, it is necessary to achieve a threshold strain prior to the initiation of bone formation.^23^ Below this strain threshold, there is an adaptive state called “lazy zone” where no net change in bone mass is observed.^121^ Results based on the four-point bending model of the rat tibia^27^ and the avian forelimb loading model^26^ suggest that the adaptive strain threshold may be similar across species. However, the adaptive strain threshold appears to be distinct across different anatomical bone sites and is affected by other factors.^27^^,^^37^^,^^74^^,^^95^, ^122^, ^123^ Specifically, cancellous bone may have a greater adaptive strain threshold than cortical bone.^74^ The adaptive strain threshold of the rat ulna increases in magnitude from the proximal to the distal end, and the strain threshold is greater in regions with higher strains.^95^ In a four-point bending model of the rat tibia, Turner et al. observed that older rats had a greater adaptive strain threshold than adult rats.^27^^,^^37^ In addition, Akhter et al.^122^ and Robling et al.^123^ suggested that the adaptive strain threshold is related to bone mineral density, although this hypothesis remains to be verified.

However, a few studies have demonstrated that the bone response to increasing strain magnitude is a continuous and essentially linear function.^26^^,^^59^^,^^124^ For instance, the experimental data reported by Rubin et al. showed that there was no “lazy zone” between the externally applied peak strain and the resulting change in bone area.^26^^,^^124^ This observation was later reported by Sugiyama et al. and Christen et al.^96^^,^^125^ However, these observations may have been confounded by other factors such as surgical invasion, disuse, and estrogen deficiency. Thus, future studies with refined experimental designs are required to provide convincing evidence supporting this perspective.

**Fig. 5:**
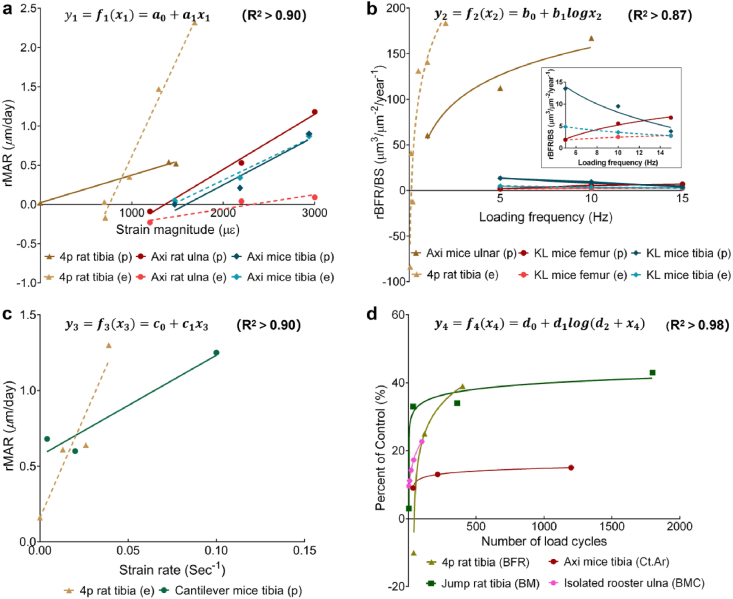
Based on outcomes of *in vivo* animal loading experiments, relationships between bone adaptive responses and mechanical loading parameters ((**a**) strain magnitude; (**b**) loading frequency; (**c**) strain rate; (**d**) number of load cycles) are determined by regression analyses (see Appendix for original data and detailed methods). Note: 4p indicates the four-point bending model; Axi represents the axial compressive loading model; KL represents the knee-loading model; p represents the periosteal surface; and e represents the endocortical surface.

Nevertheless, studies have clearly demonstrated the existence of a dose–response relationship between the peak strain magnitude and osteogenesis when the mechanically induced strain surpasses the adaptive strain threshold.^27^^,^^74^^,^^95^^,^^97^, ^98^, ^99^, ^100^ Based on the outcomes of typical *in vivo* loading studies,^27^^,^^40^^,^^95^^,^^98^ we performed regression analyses (see Appendix) and determined the following relationship between the strain magnitude and bone adaptive responses (Fig. 5a):

(1) $$y_{1}=f_{1}\left(x_{1}\right)=a_{0}+a_{1}x_{1}$$

where $y_{1}$ represents the bone adaptive changes to loading (e.g., relative mineral apposition rate [rMAR]) and $x_{1}$ is the peak strain. $a_{0}$ and $a_{1}$ are coefficients. The general form of the strain magnitude–response relationship obtained from different animal models was consistent. However, the slope and intercept of the fitted curve may depend on the animal-loading model, age, and other loading parameters.

### 3.2 Load/strain frequency

Compared with the strain magnitude, the loading frequency may affect the amount of new bone formation more substantially.^126^^,^^101^ Studies using the axial loading model of the rat ulna,^102^^,^^103^ the four-point bending model of the rat tibia,^104^ and the axial loading model of the mouse tibia^127^ have demonstrated that an increase in loading frequency leads to a dose–response increase in bone formation. However, cortical bone adaptation to mechanical loading increased with increasing loading frequency up to only 5–10 ​Hz, subsequently plateauing at frequencies beyond 10 ​Hz.^41^

Regression analyses (see Appendix) were performed based on the outcomes of several above-mentioned typical *in vivo* experimental studies.^41^^,^^104^^,^^127^ A logarithmic relationship was detected between loading frequency and bone adaptive responses. Among these studies, only one covered the frequency range of 1–30 ​Hz. Although including data from 20 to 30 ​Hz may reveal a plateau phase, this would compromise the integrity of the logarithmic relationships. Therefore, further research is required to investigate high-frequency phases. Scheuren et al. also demonstrated a logarithmic relationship between bone adaptation (e.g., relative bone volume fraction) and loading frequency, effectively showcasing the plateau phase.^105^ However, differences in bone formation parameters across studies make it challenging to obtain consistent adaptive bone response parameters. Therefore, the focus of this review is to emphasize the logarithmic relationship. Consequently, based on the existing data, we focused on presenting the logarithmic relationship rather than introducing high-frequency data that might obscure this key point (Fig. 5b).

(2) $$y_{2}=f_{2}\left(x_{2}\right)=b_{0}+b_{1}\log x_{2}$$

where $y_{2}$ represents bone adaptive changes to loading (e.g., relative bone formation rate [rBFR/BS]) and $x_{2}$ is the frequency. $b_{0}$ and $b_{1}$ are the constants.

### 3.3 Load/strain rate

The strain rate is an essential parameter for stimulating new bone formation.^38^^,^^39^ Several experiments demonstrated that the strain rate is a function of the strain magnitude and loading frequency.^87^ During normal gait, the increase in strain magnitude and frequency is reflected in the change in a single parameter of the strain rate. The strain rate also depends on the applied loading waveform (e.g., sinusoidal or trapezoidal).^128^ The strain rate and frequency can be isolated with trapezoidal or triangle loading waveforms by inserting “rest” between cycles or altering the length of time spent at the peak load.^88^^,^^106^ Sinusoidal waves can be used to separate the frequency and strain rates by changing the peak-to-peak strain values.^38^

*In vivo* loading studies have indicated that a higher strain rate can enhance bone formation when compared with low or medium strain rates. There is a dose–response relationship between the strain rate and periosteal new bone formation, whereas the adaptive response of the endosteal cortex does not increase with the strain rate.^107^ However, Turner et al. detected a linear relationship between the applied strain rate and endocortical adaptive responses.^38^ In addition, the dose–response relationship between the strain rate and bone adaptation was replicated in numerical simulations.^129^

Regression analyses were performed based on the outcomes of typical *in vivo* experimental studies^38^^,^^107^ and the relationship between the strain rate and bone-adaptive responses can be described as follows (Fig. 5c).

(3) $$y_{3}=f_{3}\left(x_{3}\right)=c_{0}+c_{1}x_{3}\;\;\;$$

where $y_{3}$ represents the bone adaptive changes to loading (e.g., rMAR), $x_{3}$ is the strain rate, $c_{0}$ and $c_{1}$ are coefficients.

### 3.4 Number of load cycles

Various load cycles have been used for *in vivo* animal loading experiments.^30^^,^^108^, ^130^, ^131^ Only a few load cycles are required to induce the adaptive response of the bone (e.g., 4–60), and the bone formation response tends to exhibit diminishing returns with an increase in the number of load cycles.^96^^,^^132^ For example, establishing the isolated ulna model in a rooster, Rubin et al. showed that increasing the number of daily load cycles from 36 to 1800 did not enhance bone formation, suggesting that the response of bone cells to mechanical loading rapidly approached saturation.^55^ Likewise, Umemura et al. found that 5 jumps/day was sufficient to increase bone mass, and a continued increase in jumping times reduced the gain of bone mass.^55^ In addition, Yang et al. and Sun et al. showed that reducing the daily cycle number sequentially from 1200 to 300 to 60 did not diminish the bone formation response.^40^^,^^55^ Interestingly, load-induced osteogenesis of cancellous bone in the mouse tibia appeared to saturate faster than that of cortical bone in response to increasing numbers of daily load cycles.^40^

Regression analyses between the number of load cycles and bone responses based on previous experimental data^40^^,^^55^^,^^133^^,^^109^ determined the following logarithmic relationship (Fig. 5d):

(4) $$y_{4}=f_{4}\left(x_{4}\right)=d_{0}+d_{1}\log\left(d_{2}+x_{4}\right)$$

where $y_{4}$ represents the bone adaptive changes to loading (e.g., bone formation rate, cortical area (Ct.Ar), bone mass (BM), and bone mineral content (BMC)); $x_{4}$ represents the number of load cycles per day; and $d_{0}$, $d_{1}$, and $d_{2}$ are coefficients. A logarithmic relationship between the load cycle number and bone response has also been reported.^40^

### 3.5 Rest insertion

Following relatively few load cycles, the adaptive response of the bone reaches saturation, and the bone cells become insensitive to any additional mechanical stimulation. The mechanosensivity of the loaded bone can be restored by dividing the entire loading period into short bouts or by inserting rest times between individual cycles. Robling et al. found an enhanced osteogenic response when 360 cycles per day were divided into four bouts of 90 cycles or six bouts of 60 cycles per day,^134^^,^^110^ with an optimal response observed when a recovery period of 4–8 ​h was allowed between the loading bouts.^135^ Srinivasan et al. used a four-point bending model of adult turkeys and found that low-magnitude cyclic loading did not alter periosteal bone formation, whereas a combination of 10 ​s rest and low-magnitude loading was osteogenic.^111^ A study using a rat four-point bending model showed that 14 ​s of short intervals resulted in a substantially higher bone formation rate than the other three short intervals (0.5, 3.5, and 7 ​s).^75^ However, contradictory findings have also been documented; for example, in a tibial axial compressive loading study, inserting a rest between every four cycles yielded no benefits.^40^ These contradictory results could be attributed to the interactive effects of the different loading parameters.^40^ For example, inserting 10 ​s of rest between individual low-magnitude load cycles could markedly enhance periosteal bone formation in older mice, whereas the insertion of short intervals does not provide additional benefits when the strain magnitude or number of load cycles is doubled.^112^ These results suggested that the osteogenic effects of rest insertion may be suppressed by high-magnitude loads or strains.^136^

In addition, the contradictory results regarding short rest intervals and bone mechanoadaptation may arise not only from the interactive effects of different loading parameters but also from limitations in the current utilization of *in vivo* animal loading models. Studies often lack reproducibility owing to excessive standardization, such as genetic standardization (animals) and environmental standardization (housing and husbandry). Voelkl et al. demonstrated that multilaboratory designs with heterogeneous samples can improve reproducibility when compared with single-laboratory studies.^137^ Furthermore, challenges in standardizing protocols complicate the interpretation of results across different studies.^138^^,^^139^ Therefore, addressing these limitations is essential to advance our understanding of the precise relationship between mechanical signals and bone adaptation.

### 3.6 Loading waveform

The bone response to loading is sensitive to the loading waveform.^106^ Sinusoidal,^58^ trapezoidal,^140^ triangular,^79^ and sawtooth waveforms^67^ have been used in *in vivo* animal-loading studies. To our knowledge, no experimental studies have directly compared osteogenic responses between different loading waveforms, although computational studies have provided insights into this issue.^141^^,^^142^ Recently, finite element studies of the cortical bone simulating the gait loading waveforms of walking, running, and jumping indicated that only running can improve the pore pressure and fluid velocity in healthy and osteoporotic tissues.^141^ Kumar et al. conducted a numerical simulation using a cantilever-bending model of a mouse tibia to compare the pore pressure and poroelastic responses produced by three different non-sine waves.^142^ The authors found that, compared with sawtooth and triangular loading waveforms, trapezoidal loading generated the maximum pore pressure and canalicular fluid velocity.^142^

The strain rate, frequency, and magnitude of the loading waveform are interrelated. Turner proposed that the strain rate is proportional to frequency and strain magnitude. Turner^87^ assumed that the strain stimulus is proportional to the strain rate based on three rules for bone adaptation to mechanical stimuli, expressing the relationship between the strain stimulus and any periodic loading waveform using the Fourier transform method. This method allows any periodic loading waveform to be decomposed into the sum of sine waves at different strain magnitudes and frequencies. Consequently, by adding the contributions of the n significant frequency components of the waveform, the strain stimulus of any periodic waveform can be expressed as follows:

(5) $$E=k_{1}\sum_{i=1}^{n}x_{1_{i}}x_{2_{i}}\;\;$$

where E is the strain stimulus, $x_{1}$ is the peak strain, $x_{2}$ is the loading frequency, n is the number of significant frequency components, and $k_{1}$ is a proportionality coefficient.

### 3.7 Interactions between different loading parameters

Previous experimental studies have indicated that distinct loading parameters have interactive effects on bone response. Rubin et al. found that dynamic loading with high-frequency (20–50 ​Hz) and extremely small strains (<10 με) exerted a positive effect on the cancellous bone formation of adult sheep.^143^ At a constant frequency, the number of load cycles required to activate formation depends on the strain magnitude.^133^ For example, a high level of strain can markedly induce an osteogenic response in a short time, whereas a prolonged medium-level strain can also be osteogenic.^113^ However, if a sufficient number of load cycles are applied at an appropriately high frequency, extremely low strain levels can prevent bone loss in the disused state.^144^ Qin et al. established a nonlinear relationship between the number of load cycles per day and the peak strain magnitude for bone mass maintenance by curve-fitting the results of several *in vivo* experimental studies.^114^

(6) $$x_{1}=10^{2.28}{\left(5.6-\log_{10}x_{4}\right)}^{1.5}$$

where $x_{1}$ represents the peak strain and $x_{4}$ is the load cycle number per day.

Interactions between rest insertion and other loading parameters also need to be considered. For instance, Srinivasan et al. demonstrated that 50 low-magnitude load cycles per day were not osteogenic, but inserting 10 ​s of rest between individual cycles could substantially enhance the periosteal bone formation rate.^112^ Additionally, using the tibia cantilever bending model, LaMothe et al. found that, compared with continuous loading of 30 ​Hz, inserting a 10 ​s rest period could markedly enhance the periosteal osteogenesis rate, although the total number of load cycles was reduced by 10-fold.^115^ Moreover, rest insertion can reduce the number of loading bouts required to enhance bone formation^116^ and rapidly amplify the response of the bone to small increases in strain and load cycles.^117^ Recently, a multiscale modeling study demonstrated that strain magnitude and rate were the main factors affecting the velocity and fluid shear stress in the lacunar-canalicular system of osteocytes, identifying interactions between strain magnitude, rate, and rest insertion.^145^

In summary, we provide a comprehensive review of the quantitative relationships between different mechanical signals and the adaptive responses of bone, which have been identified based on the outcomes of various *in vivo* loading studies. Bone mechanoadaptation is a complex and dynamic mechanobiological process influenced by various pathophysiological factors.

## 4 Factors affecting the relationships between mechanical signals and bone adaptation

**Fig. 6:**
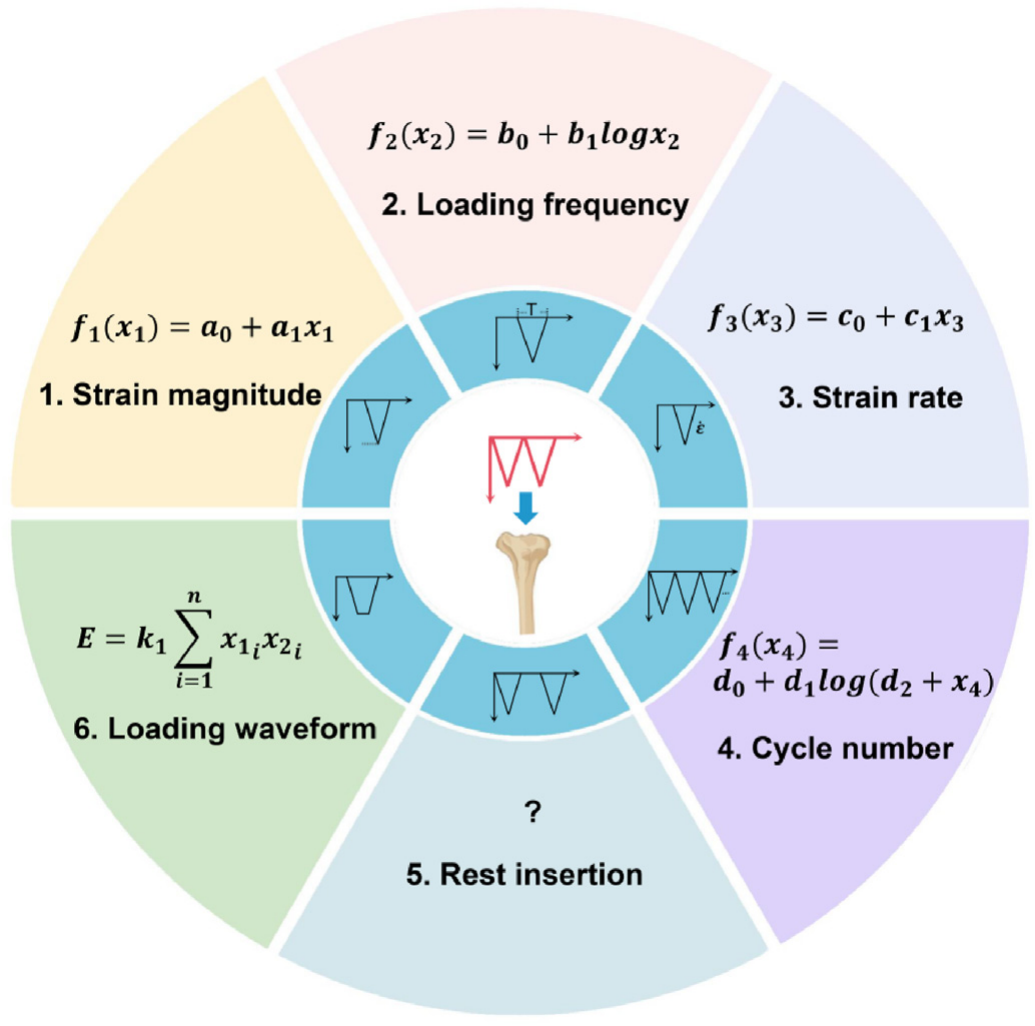
The relationships between mechanical loading parameters (e.g., strain magnitude, loading frequency, strain rate, number of load cycles, rest insertion, and loading waveform) and bone adaptive responses, including some that have been identified according to previous animal loading experiments and others that need to be determined.

In this section, we focus on the important pathophysiological factors that may influence the relationship between mechanical signals and bone adaptive responses, such as genetics, sex, age, disuse, estrogen deficiency, and circadian rhythm. These factors could alter the strain *‘setpoint’* (i.e., minimum effective strain) as illustrated in Frost's mechanostat diagram (Fig. 2b) and also modify the pre-identified relationships (Fig. 6). Here, we briefly review relevant studies and findings that may be valuable in designing effective mechanical regimens to improve bone health in the context of aging, disuse, and other circumstances.

### 4.1 Genetics

Experimental studies have found that the daily strain history is similar for bones of different species, with greater strains (>1000 με) occurring less frequently and very low strains (<∼200με) occurring thousands of times per day.^146^ However, changes in bone mineral density or gene expression induced by mechanical loading differ substantially in inbred mice.^147^^,^^148^ This implies that genetic components govern the osteogenic response of bone to mechanical loading.^58^^,^^123^^,^^149^

### 4.2 Sex

The response of bones to mechanical loading is sex-specific.^150^, ^151^, ^152^ Under the same peak strain of 2500 με, young female mice (19-week-old) gained more cortical bone than males in specific regions (e.g., around 37 ​% site) of the tibia, although these site-specific sex-dependent differences vanished with age (19-month-old).^153^ However, when similar levels of peak strain are engendered in males and females, the adaptive response of cancellous bone to mechanical loading does not depend on sex in growing mice.^154^ Factors affecting the differential loading responses of bones between males and females may include differences in estrogen and androgen receptors, bone mass, and structure.^155^^,^^156^

### 4.3 Age

According to the findings of an axial compression loading study, the deformation pattern and orientation of the neutral axis did not differ considerably between the tibiae of young adult and older mice.^157^ However, the bone appears to become less sensitive to mechanical loading with age^85^^,^^158^, ^159^, ^160^, ^161^, ^162^, ^163^ (Table 3). Birkhold et al. highlighted that mechanical responses within the endocortical bone surface continued to decline with age, whereas the main decay in the periosteal surface formation response occurred before adulthood.^85^ Subsequently, the authors found that mice in all age groups (young, adult, and older) responded to high strain by increasing bone formation and decreasing absorption, whereas low strain only had a strong osteogenic effect on young mice.^158^ Similarly, cancellous bone response to *in vivo* loading decreases with age.^164^^,^^165^ Contrary to these findings, several experimental studies found no difference in the mechanoresponse of cortical bone between young and adult mice.^76^^,^^166^ The adaptive responses of growing mice to mechanical loading may also be influenced by their growth rate.^152^ Collectively, these results indicate that the mechanical adaptability of the bone in older mice is substantially reduced. However, there is no consensus regarding how age affects bone mechanoadaptation prior to maturity.

**Table 3:** Experimental studies examining the influence of age on bone adaptation to mechanical loading.

Reference	Loading model	Animal model	Age	Bone types	Key findings
Turner et al.^37^	Four-point bending	Rat tibia	9 and 19 months	Cortical	The relative bone-forming surface in old rats appears 5-fold less than in younger rats under similar loading conditions, with no significant differences observed in the formed woven bone.
				Woven	
De souza et al.^76^	Axial compression	Mouse tibia	8, 12, and 20 weeks	Cortical	Loading markedly increases cancellous bone volume in 8-week mice but decreases at 12 and 20 weeks, while cortical bone formation appears similar.
				Cancellous	
Birkhold et al.^85^	Axial compression	Mouse tibia	10, 26, and 78 weeks	Cortical	The osteogenic response of the periosteal surface and endosteal surface declines with the increase in mouse age.
Carriero et al.^119^	Axial compression	Mouse tibia	10 and 22 weeks and 20 months	–	The magnitude of strain at a given load generally increases with age.
Patel et al.^157^	Axial compression	Mouse tibia	5, 12, and 22 months	–	The deformation pattern and orientation of the neutral axis do not differ substantially in the tibia between young adult and older mice.
Razi et al.^158^	Axial compression	Mouse tibia	10, 26, and 78 weeks	Cortical	The response of mice in all age groups to high strains occurs through increased formation and decreased absorption, while low strains only induce strong responses in young mice.
Holguin et al.^159^	Axial compression	Mouse tibia	5, 12 and 22 months	Cortical	The young adult mice show greater periosteal bone formation following loading than middle-aged or old mice.
				Cancellous	
				Woven	
Birkhold et al.^160^	Axial compression	Mouse tibia	10, 26, and 78 weeks	Cortical	Loading exerts a substantially stronger effect on formation than on resorption, which is mainly due to an increase in surface area formation with loading.
Birkhold et al.^161^	Axial compression	Mouse tibia	10, 26, and 78 weeks	Cortical	Increased surface formation with mechanical stimulation is the only effect of loading, which is conserved into old age.
				Cancellous	
Rubin et al.^162^	Surgically-isolated ulnar loading	Turkey ulna	1 and 3 years	Cortical	A physical signal that is clearly osteogenic in the young adult skeleton is hardly recognized in older bone tissue.
Lambers et al.^163^	Vertebral compression	Mouse sixth caudal vertebra	15, 52, and 82 weeks	Vertebrae	The mechanical sensitivity of mouse vertebrae is maintained with age.
Meakin et al.^164^	Axial compression	Mouse tibia	17 weeks and 19 months	Cortical	In adulthood, increasing age is associated with a reduction in adaptive responses of cortical and cancellous bones to loading in both male and female mice.
				Cancellous	
Lynch et al.^165^	Axial compression	Mouse tibia	10 and 26 weeks	Cortical	The cancellous bone in the adult skeleton remains responsive to mechanical loading but requires greater osteogenic stimuli than a younger skeleton.
				Cancellous	
Willie et al.^166^	Axial compression	Mouse tibia	10 and 26 weeks	Cortical	No difference can be observed in the mechanical reactivity of tibial cortical bone between young and adult mice, while the response of cancellous bone in adult mice to load is reduced and delayed than that in young mice.
				Cancellous	

With aging, the balance in mechanoregulated bone remodeling leads to reduced bone formation and enhanced bone resorption, eventually leading to a decrease in bone mass.^167^^,^^168^ Mechanical loading can increase cortical bone mass by shifting bone formation and resorption balance toward bone formation. Recently, Javaheri et al. proved that mechano-adaptive responses of aged bones are insufficient (although relatively high strain is induced) but can be restored by applying disuse or supra-physiological high-magnitude loads.^169^ The authors also proposed a new concept suggesting that the mechanoadaptation of aged bone is more sensitive to large strain gradients than to strain magnitude,^169^ providing new insights for the design of innovative exercise paradigms to combat age-related bone loss.

### 4.4 Disuse

Disuse-induced osteoporosis describes a state of bone loss due to local or systemic skeletal unloading (e.g., long-term bed rest,^170^ immobilization,^171^ paralysis,^172^ and spaceflight^173^^,^^174^). Disuse-induced bone loss follows a remodeling process similar to that typically occur during aging or osteoporosis.^175^ Disuse promotes the recruitment of osteoclasts to the bone surface for rapid bone resorption. Osteoblast precursors migrate to the bone surface and differentiate into osteoblasts, initiating bone formation. The main cause of bone loss is a reduction in mechanical loading.^176^ Preclinical rodent hindlimb unloading^177^ and sciatic neurectomy models^32^^,^^178^ have been commonly used to simulate disuse conditions and to examine how disuse affects bone mechano-responsiveness by applying extra *in vivo* mechanical loading to the disused bone. Interestingly, disused bone shows an enhanced response to loading.^153^^,^^178^, ^179^, ^180^, ^181^ However, recent experimental studies have shown that the mechanosensivity of the tibia to reloading is substantially compromised in mice previously exposed to disuse.^182^^,^^183^ Nevertheless, the mechanisms underlying these observations remain unknown and warrant further investigation.^184^

### 4.5 Estrogen deficiency

Estrogen affects bone mass by regulating the balance between bone formation and resorption.^185^ Estrogen deficiency causes a reduction in bone mass and rapid deterioration of the cancellous microstructure, leading to osteoporosis and increased fracture risk.^186^^,^^187^ Ovariectomy has been widely used to investigate the role of estrogen in the mechanical adaptation of the bone. *In vivo* studies have revealed that ovariectomy-induced estrogen loss increases the response of bones to mechanical loading and exercise.^188^^,^^189^ Low-dose estrogen treatment was shown to suppress periosteal bone formation in response to mechanical loading.^190^

### 4.6 Circadian rhythm

The bone absorption marker C-terminal telopeptide of type I collagen, the bone mineralization marker (produced by osteoblasts and osteocalcin), and the phosphate metabolic regulator FGF-23 secreted by osteoblasts are well-known to exhibit a circadian rhythm.^191^, ^192^, ^193^ These three proteins increased overnight, reaching their peak in the morning and their lowest levels in the late afternoon.^193^^,^^194^ In addition, nearly half of the genes in the mouse genome oscillate with the circadian rhythm in the body.^195^ Importantly, there is an inevitable relationship between life rhythms and bone remodeling. Daytime exercise schedule affects the physiology and rhythmicity of gene expression in the adrenal gland, liver, and heart.^196^ A recent experimental study confirmed that circadian rhythms can affect bone mechano-responsiveness and gene expression characteristics.^169^^,^^197^ Loading at zeitgeber time (ZT) 14 resulted in larger endocortical bone formation than that at ZT 2.^197^

### 4.7 Other factors

The adaptive response of the bone to loading is affected by other factors, such as the intake time of food and fat content. A correlation between the time of food intake and the adaptive response of the bone to mechanical loading has been reported.^140^ To study whether obesity affects the bone response to loading, Eller et al. induced the same level of tibial strain in high-fat diet (HFD)- and normal diet (ND)-fed mice.^198^ The bones of HFD and ND mice responded similarly to loading, suggesting that HFD minimally impacted bone mechanoadaptation. In addition, Lagerquist et al. induced adipocyte apoptosis in male mice using the AP20187 dimer and found that acute reduction of fat did not affect bone mass in the short term.^199^

## 5 Cellular regulation of relationships between mechanical signals and bone adaptation

### 5.1 The process of bone mechanoadaptation

**Fig. 7:**
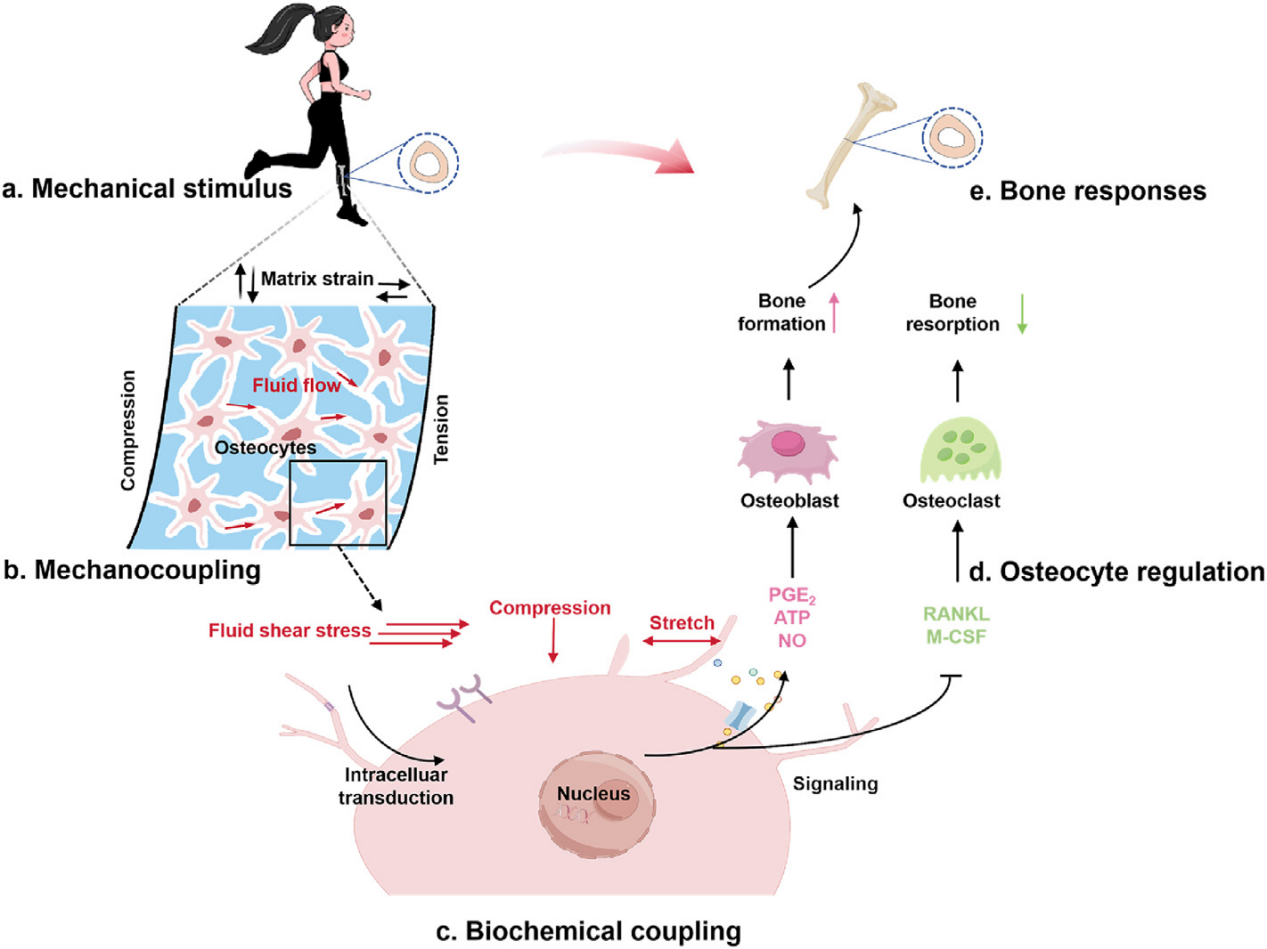
The process of bone adaptation to mechanical loading. Apparent-level mechanical forces (**a**) produce a micromechanical environment (e.g., interstitial fluid flow) for mechanosensitive cells (osteocytes) in bone (**b**). Osteocytes sense micromechanical signals and produce biochemical signals (**c**), which regulate the functions of osteoblasts and osteoclasts. Bone formation and resorption executed by osteoblasts and osteoclasts (**d**), respectively, leading to altered mass and morphology of the bone (**e**).

Bone adaptation to loading involves mechanotransduction at multiple scales,^200^ allowing bones to modify their shape and internal structure according to changes in the external mechanical environment to provide maximum strength with minimal mass (Fig. 7). Specifically, mechanotransduction in bone must include four consecutive phases:^201^^,^^202^ (1) *mechanocoupling*, the transduction of mechanical force applied to the bone into a local mechanical signal perceived by a sensor cell (e.g., osteocytes); (2) *biochemical coupling*, the transduction of a local mechanical signal into biochemical signals and, ultimately, gene expression; (3) *osteocyte regulation*, signal transmission from mechanosensor cells to effector cells (i.e., osteoblasts and osteoclasts); and (4) *the effector cell response*, bone formation and resorption executed by osteoblasts and osteoclasts, respectively.

### 5.2 Mechanosensitive cells and mechanoreceptors

Bone cells include osteoblasts, osteoclasts, osteocytes, and their progenitors, all of which can more or less respond to mechanical stimuli.^184^^,^^200^ Osteocytes, comprising over 90–95 ​% of all bone cells, are widely accepted as primary mechano-sensing cells within the bone.^203^ Additionally, osteocytes play a critical role in regulating osteoblasts and osteoclasts.^200^^,^^204^ When bones are subjected to external mechanical stimuli, osteocytes can work in conjunction by signaling osteoclasts and osteoblasts to optimize bone mass and structure.^201^

**Fig. 8:**
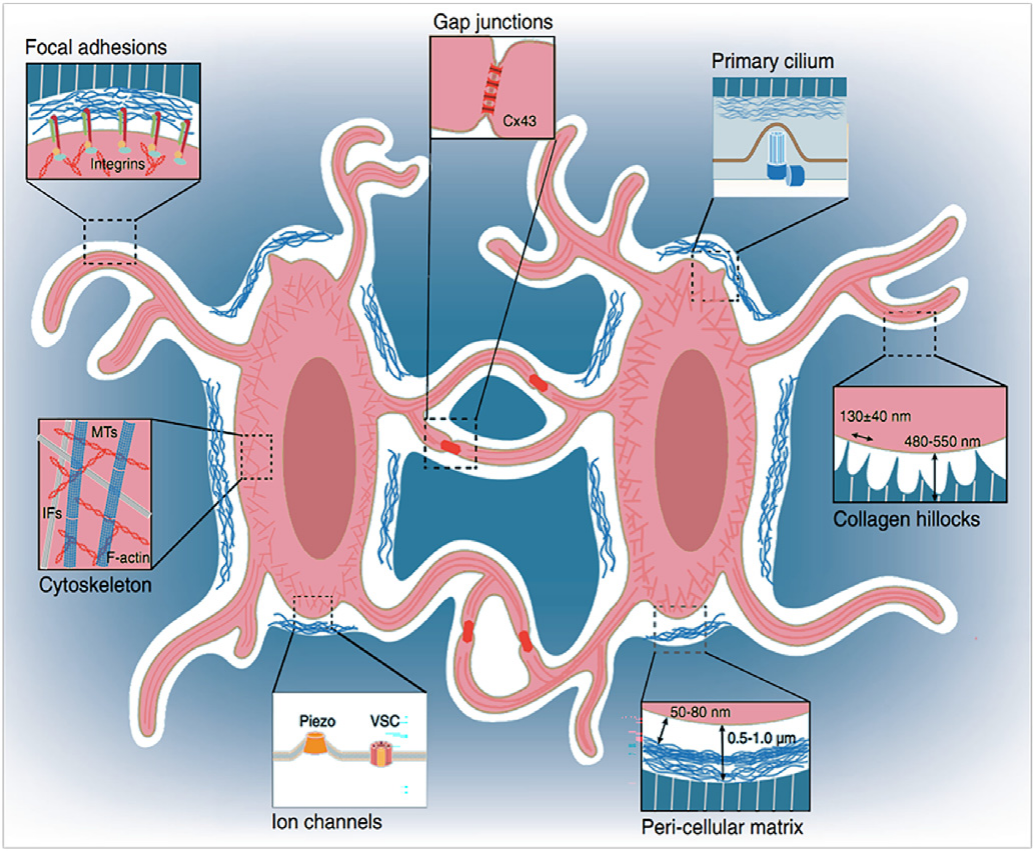
Mechanical loading (e.g., bending forces) produces interstitial fluid flow within the lacunar-canalicular system (LCS), which is sensed by mechanoreceptors of osteocytes. The figure was adapted and modified from.^206^

Osteocytes are embedded within the mineralized bone matrix, their cell bodies are housed in lacunae, and their cell processes are located in narrow canals, the canaliculi. This system, comprising both lacunae and canaliculi, is termed the osteocyte lacunar-canalicular system (LCS) and serves as the central functional unit for the transduction of apparent-level mechanical forces into the cellular mechanical microenvironment (e.g., fluid flow and shear stress) of osteocytes^205^ (Fig. 7b). Mmechanoreceptors in osteocytes include focal adhesions, gap junctions, primary cilium, cell cytoskeleton, ion channels (such as voltage-sensitive calcium channels, ionotropic channels, and mechano-sensitive channels), peri-cellular matrix in the lacunar region, and collagen hillocks in the canalicular region^206^^,^^207^ (Fig. 8).

### 5.3 Mechanical microenvironments of osteocytes

Osteocytes can respond to cellular mechanical stimuli, including matrix deformation, fluid flow-induced fluid shear stress, hydrostatic pressure, streaming potentials, and the drag force of tether fibers.^208^ However, several experimental and numerical studies have shown that interstitial fluid flow is the primary mechanical stimulator of osteocytes.^208^, ^209^, ^210^, ^211^ When a long bone is loaded, pressure gradients are created throughout the bone tissue and drive fluid flow through the LCS, generating shear stress or drag forces on the osteocytes.^212^^,^^213^ Various mechanoreceptors in osteocytes can sense extracellular mechanical signals and transduce them into biochemical signals, which require responsive gene expression to regulate the behavior of osteoblasts and osteoclasts.^214^ Moreover, load-induced fluid flow may affect the solute transport of signaling molecules (e.g., such as nitric oxide, prostaglandin E_2_, and adenosine triphosphate) between osteocytes and effector cells.^215^

### 5.4 Effects of different loading parameters on the mechanical microenvironment of osteocytes

**Fig. 9:**
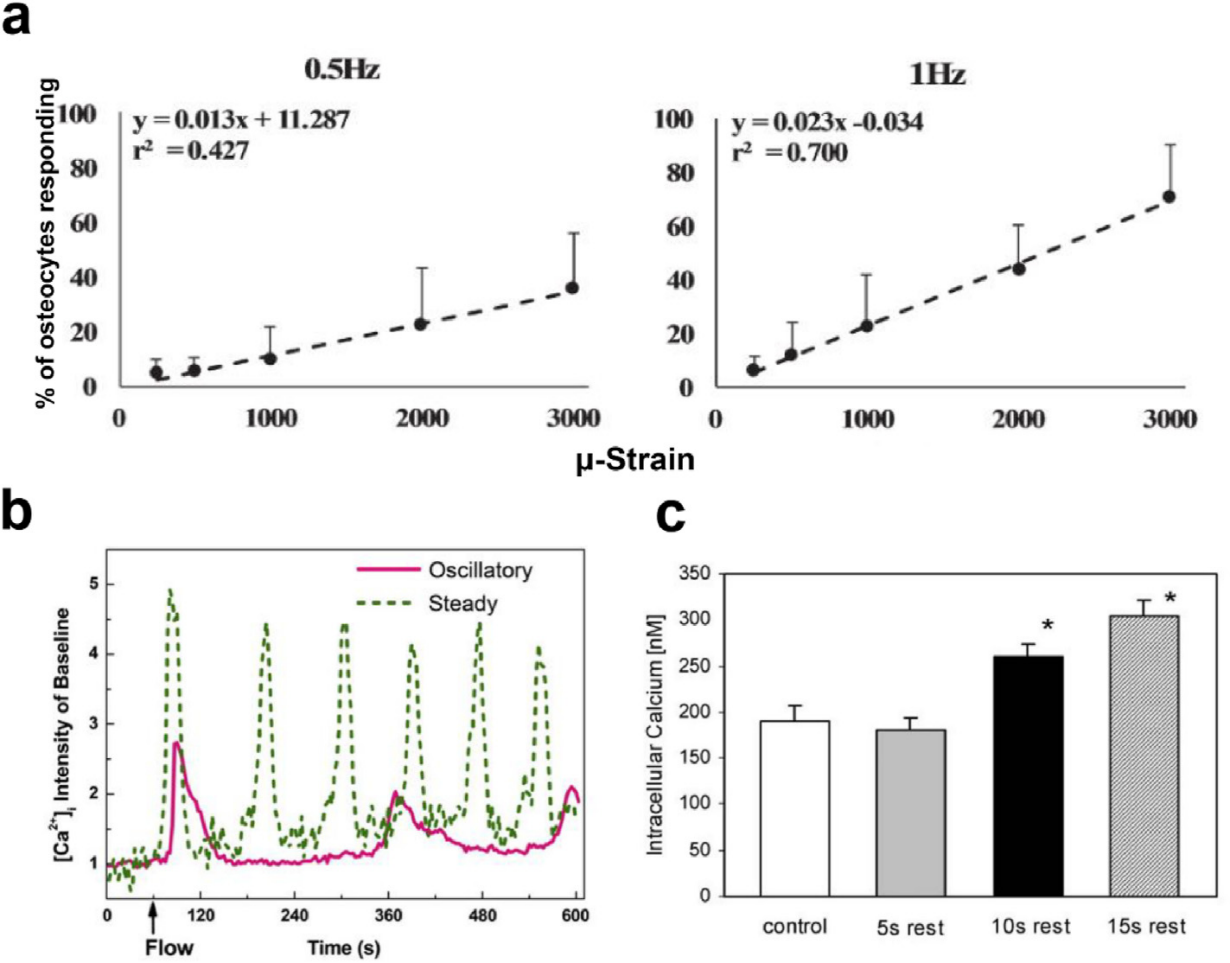
The effect of different mechanical signals on the response of bone cells. (**a**) The percentage of responding osteocytes as a function of applied strain magnitude (adapted from ^216^). (**b**) [Ca^2+^]_i_ responses of MLO-Y4 cells under steady and oscillatory loading (adapted from ^217^). (**c**) Insertion of rest (e.g.,10 and 15 ​s) into the loading profile results in higher peak response magnitudes of Ca^2+^ (adapted from ^219^).

*In vivo* experiments provided direct evidence of bone cell responses to loading, revealing that the number of responsive osteocytes increased strongly with increasing strain magnitude^216^ (Fig. 9a). However, as loading continued, the number of responsive cells gradually decreased, and even those that remained responsive displayed smaller spikes in intracellular calcium ([Ca^2+^]_i_)^217^ (Fig. 9b). Moreover, *in vitro* experiments showed that the insertion of rest intervals may recover cell sensitivity and substantially increase the Ca^2+^ transient^218^^,^^219^ (Fig. 9c).

Other studies have utilized mathematical modeling to examine the effects of various loading parameters on fluid dynamics in the LCS. Wu et al. found that the fluid flow rate and shear stress within the canaliculi of a loaded osteon were proportional to the strain magnitude and frequency.^129^ Kumar et al. established a new mathematical model of an LCS with curved canalicular walls, revealing that the peak flow velocity increased with increasing loading frequency. As the number of load cycles increased, the canalicular flow velocity was maximal during the first load cycle and reached steady-state levels after the 4th load cycle of the regimen.^220^ These numerical results were generally consistent with those observed in *in vivo* animal loading experiments.

**Fig. 10:**
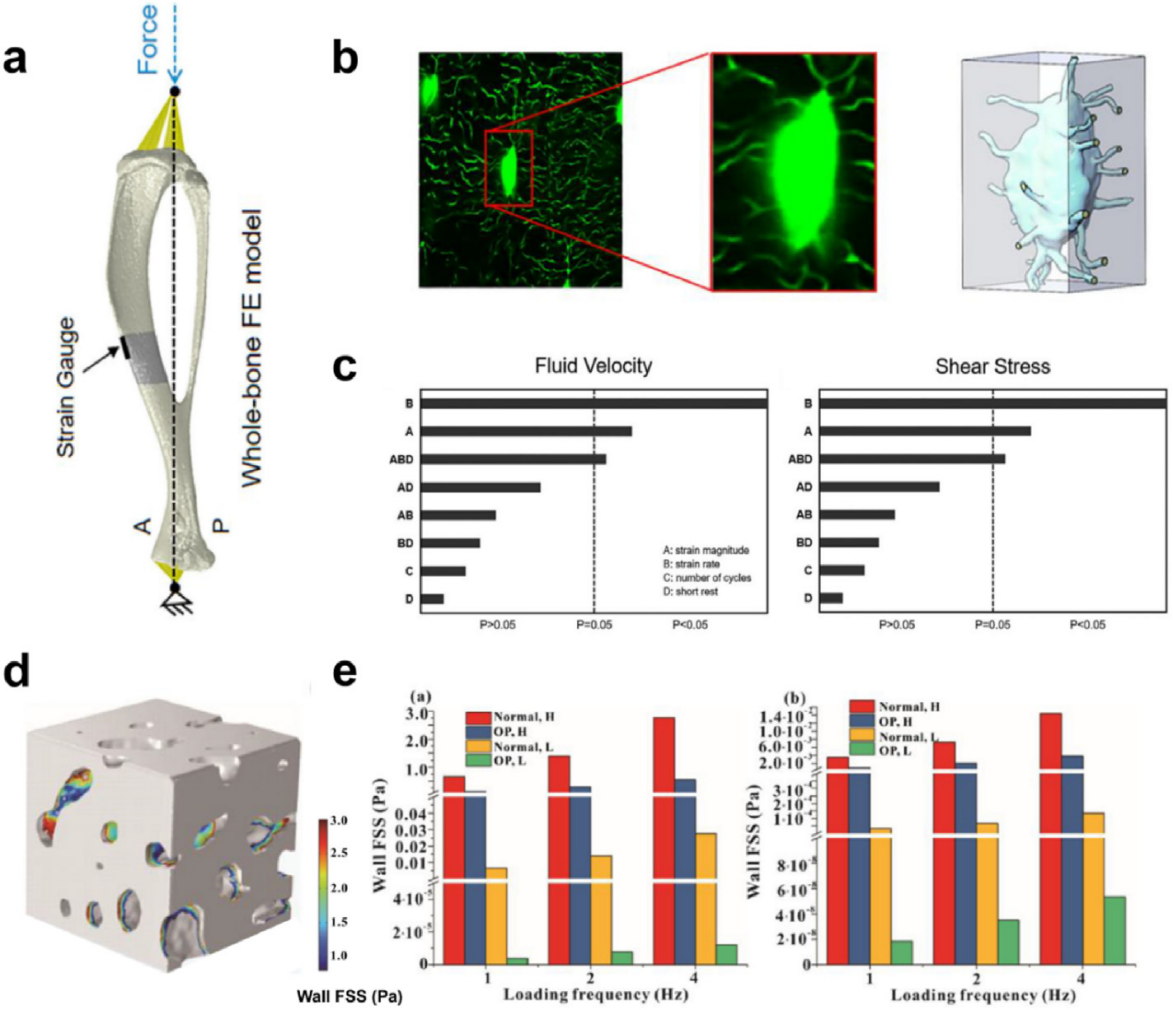
The effects of various loading parameters on the fluid dynamics around osteocytes (adapted from ^145,224^). (**a**) The whole-bone finite element model of the tibia. (**b**) A three-dimensional model of the osteocyte lacunar-canalicular system (LCS) created from confocal images. (**c**) Pareto chart for the fluid velocity and shear stress within the inlet canaliculi. (**d**) Finite element model of trabecular bone cube. (**e**) Mean fluid shear stress on osteocyte processes (left) and osteocyte surface (right).

More recently, some studies have developed multiscale models to explore the effects of different loading parameters on fluid dynamics in the porous structures of bones (such as the LCS and trabecular-lacunar cavities)^221^, ^222^, ^223^, ^224^, ^225^, ^226^ (Fig. 10). Fan et al. found that increasing the magnitude of the applied load enhanced the fluid flow within the LCS.^213^ Similarly, the fluid shear stress on osteocytes showed a loading-frequency-dependent phenomenon, and the fluid shear stress in the trabecular pores exhibited a linear increase with the loading frequency.^224^^,^^227^ Wang et al. established a multiscale model of “whole bone-single osteocyte LCS” to examine the interactive effects of various loading parameters on fluid dynamics around the osteocytes (Fig. 10a and b). The authors demonstrated that either the strain magnitude or rate was the main factor affecting the flow velocity and shear stress, and distinct loading parameters had highly interactive effects.^145^ These results are consistent with the quantitative relationships between mechanical signals and bone responses identified in animal loading studies, suggesting that fluid dynamics within the LCS may provide a new pathway, other than tissue-level strain, to decode the complex relationships between different mechanical signals and bone adaptive responses.

## 6 Summary and future perspectives

**Fig. 11:**
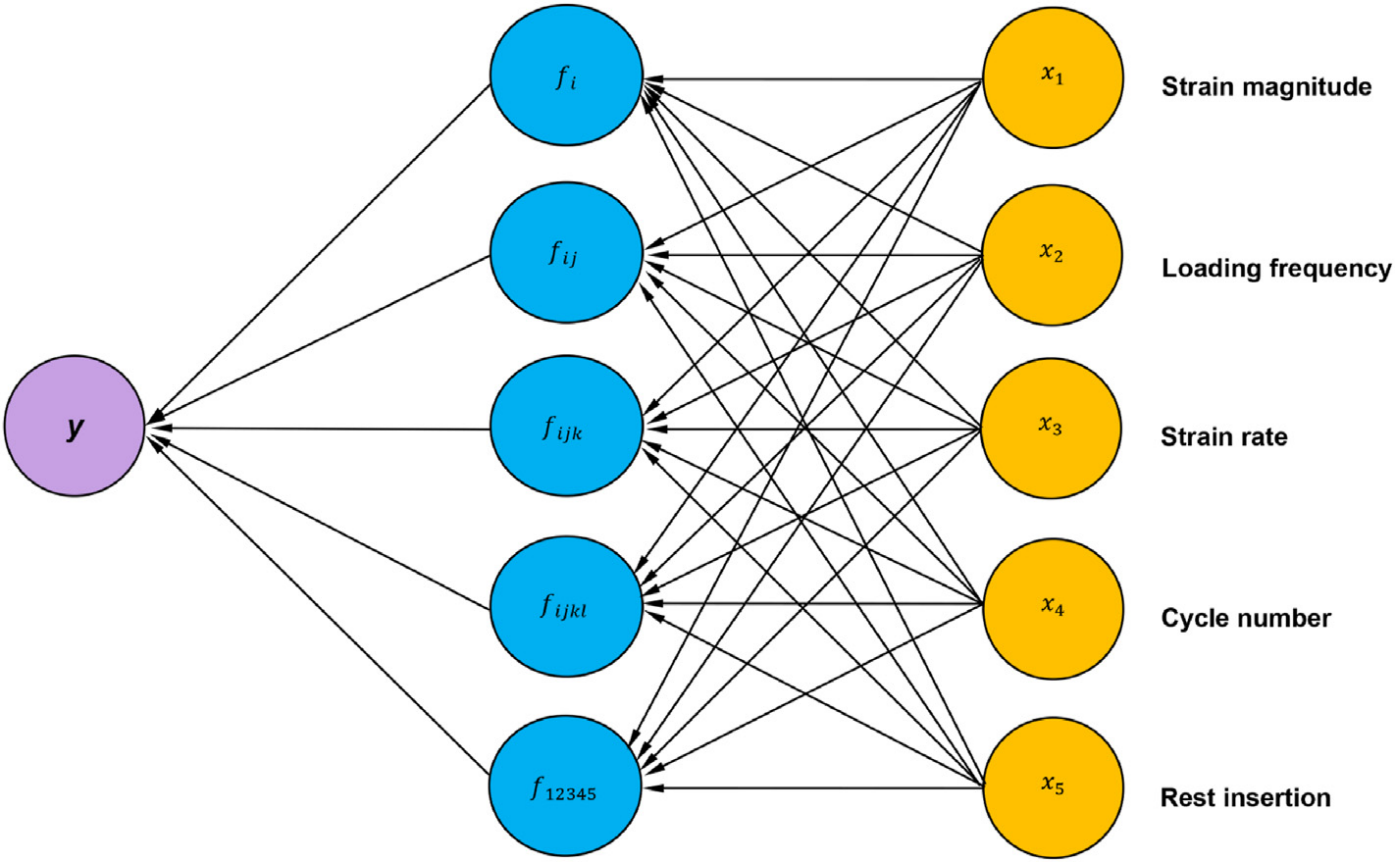
Bone adaptative responses (*y*) as functions of various loading parameters (*x*_1_-*x*_5_), when other parameters are constant. *y* represents the bone adaptive changes to loading. *x*_1_, *x*_2_, *x*_3_, *x*_4_, and *x*_5_ represent the strain magnitude, loading frequency, strain rate, number of load cycles, and rest insertion, respectively.

Bone adaptation to mechanical loading has long been recognized and elaborated upon by Julius Wolff in his book *The Law of Bone Remodeling*.^228^ Furthermore, “*The Mechanostat”* theory by Frost suggests that strain is the primary mechanical stimulus governing bone adaptation.^229^ However, *in vivo* animal loading experiments revealed diverse bone responses to different loading parameters, even when the strain was maintained at the same magnitude. The precise relationship between mechanical stimuli and bone adaptation is complex and remains elusive. By summarizing the outcomes of relevant *in vivo* animal loading studies, we attempted to identify any known quantitative relationships between the loading parameters and bone responses to provide a better understanding of bone mechanoadaptation (Fig. 6). Based on these quantitative relationships, a general mathematical model of bone adaptation as a function of different loading waveform parameters has been proposed (Fig. 11).

The relationships between the individual loading parameters (except for the rest of the insertion) and bone responses are evident (Fig. 6). However, the effects of two or more loading parameters on bone adaptation remain largely unknown. A simple law may explain the complex phenomenon of bone adaptation to various mechanical signals. The Fourier transform can be used to generalize the strain stimulus of any periodic waveform,^87^ as reported by Turner [Equation (5)]. However, this equation has certain limitations because it does not account for interactions between the strain stimulus, number of load cycles, and rest insertion. Inspired by Turner's viewpoint and the method of incorporating the influence of cycle number,^87^ we further accounted for the effect of rest insertion into the model. A combination of Equations (4), (5) with the function $f\left(x_{5}\right)$ yields a general formula to describe the bone adaptive responses as a function of various loading parameters:

(7) $$y=k_{1}\log\left(d_{0}+x_{4}\right)f\left(x_{5}\right)\sum_{i=1}^{n}x_{1_{i}}x_{2_{i}}$$

where $x_{1}$ represents the peak strain, $x_{2}$ represents the loading frequency, $x_{4}$ represents the number of load cycles per day, $x_{5}$ represents the inserted rest, n represents the number of significant frequency components, $d_{0}$ represents a coefficient, and $k_{1}$ represents the proportionality coefficient.

However, the relationships between mechanical signals and bone adaptation have mostly been identified based on *in vivo* animal loading experiments, while their underlying mechanism remains unknown and cannot be explained by classic theories such as *The* *Mechanostat* theory. Interestingly, recent studies have provided new insights into how mechanical stimuli influence bone adaptation through numerical simulations^230^, ^231^, ^232^, ^233^, ^234^ and have attempted to employ neural network models to establish empirical relationships between loading parameters and bone responses (e.g., mineral apposition rate).^235^^,^^236^

Given that tissue-level strain cannot explain all experimental observations and bone adaptation is executed by bone cells, new mechanistic insights may be offered at the cellular level. Osteocytes sense the mechanical stimuli and regulate bone formation and resorption. The bone response may be directly related to the intensity of the mechanical microenvironment of osteocytes. The LCS is a remarkable structure that transforms and normalizes different forms of apparent-level mechanical signals into the same form of fluid flow for osteocytes within the LCS. Thus, different individual loading parameters or their combinations are reflected by the altered intensity of the fluid dynamics (e.g., flow velocity and shear stress) within the LCS. For example, changes in the loading frequency or rate can lead to increases in the flow velocity in the LCS. This could provide a new pathway for elucidating the complex relationships between different mechanical signals and bone adaptation. Indeed, fluid dynamics analyses of the LCS have explained several experimental observations regarding bone adaptations induced by different mechanical signals.^129^^,^^220^, ^225^ Additionally, the morphology of the LCS is altered by pathophysiological factors such as aging, estrogen deficiency, and disuse, leading to changes in the mechanical microenvironment within the LCS and ultimately affecting bone mechanoadaptation.^226^ This may also explain why and how bone mechanoadaptation is altered by pathophysiological factors. Taken together, the complex relationship between mechanical signals and bone adaptation can be simplified from the perspective of a cellular mechanical microenvironment. Despite existing multiscale computational models prohips.viding notable insights into the relationship between mechanical signals and bone adaptatio,^129^^,^^142^^,^^145^^,^^237^ further mechanistic studies are needed to elucidate these relations.

Although bone is mechanical adaptive, not all forms of exercise are equally effective at eliciting an osteogenic response. This review describes the *in vivo* animal loading experiments and provides deeper insights into the relationship between mechanical signals and adaptive bone responses. This study may offer theoretical guidance for optimizing exercise regimens, rehabilitation strategies, and the design of medical devices to promote bone health and treat osteoporosis. This is consistent with recent studies showing that high-frequency training is beneficial for increasing bone mineral density, and tailored whole-body vibration therapies have been proven effective in improving bone density.^238^^,^^239^ Additionally, Equation (7) can be used within physiological ranges to identify the optimal mechanical stimuli for maximizing bone health, thereby guiding the design of exercise regimens and medical devices. Future research could incorporate human exercise studies to further validate and apply this formula and determine the effectiveness of various exercise modalities and training doses for more targeted interventions to enhance bone health, particularly in preventing osteoporosis.

## CRediT authorship contribution statement

**Chenlu Wang:** Writing – original draft, Investigation, Data curation. **Ruisen Fu:** Validation, Investigation. **Haisheng Yang:** Writing – review & editing, Conceptualization.

## Ethical approval

This study does not contain any studies with human or animal subjects performed by any of the authors.

## Declaration of competing interest

The authors have no conflict of interest.

## Figures and Tables

**Table:** 

No.	Strain magnitude (με)	rMAR (μm/day)	Endosteum/periosteum surfac	Loading form	Animal model	Reference
1	701	0.03	Endosteum	Four point bending	Rat tibia	Turner et al.^240^
	711	−0.17				
	975	0.35				
	1295	1.47				
	1688	2.32				
2	0	0.02	Periosteum	Four point bending	Rat tibia	Turner et al.^240^
	1404	0.54				
	1484	0.52				
3	1200	−0.23	Endosteum	Axial compression	Rat ulna	Hsieh et al.^241^
	2200	0.04				
	3000	0.09				
4	1200	−0.09	Periosteum	Axial compression	Rat ulna	Hsieh et al.^241^
	2200	0.53				
	3000	1.18				
5	1469	0	Periosteum	Axial compression	Mice tibia	Yang et al.^242^
	2183	0.21				
	2939	0.9				
6	1469	0.05	Endosteum	Axial compression	Mice tibia	Yang et al.^242^
	2183	0.34				
	2939	0.87				
**No.**	**Loading frequency (Hz)**	**rBFR/BS (μm**^**3**^**/μm**^**2**^**/year)**	**Endosteum/periosteum surfac**	**Loading form**	**Animal model**	**Reference**

1	0.05	−83.51	Endosteum	Four point bending	Rat tibia	Turner et al.^243^
	0.1	40.93				
	0.2	−12.08				
	0.5	131.25				
	1	140.958				
	2	183.63				
2	1	60.5	Periosteum	Axial compression	Mice ulnar	Warden et al.^244^
	5	112.4				
	10	167				
3	5	4.87	Endosteum	Knee loading	Mice tibia	Zhang et al.^245^
	10	3.60				
	15	2.84				
4	5	13.51	Periosteum	Knee loading	Mice tibia	Zhang et al.^245^
	10	9.52				
	15	3.83				
5	5	1.82	Endosteum	Knee loading	Mice femurr	Zhang et al.^245^
	10	2.54				
	15	2.81				
6	5	1.93	Periosteum	Knee loading	Mice femur	Zhang et al.^245^
	10	5.58				
	15	6.90				
**No.**	**Strain rate (sec**^**−**^**^1^)**	**rMAR (μm/day)**	**Endosteum/periosteum surfac**	**Loading form**	**Animal model**	**Reference**

1	0	0.16	Endosteum	Four point bending	Rat tibia	Turner et al.^246^
	0.013	0.61				
	0.026	0.64				
	0.039	1.30				
2	0.004	0.68	Periosteum	Cantilever bending	Mice tibia	LaMothe et al.^247^
	0.02	0.60				
	0.1	1.25				
**No.**	**Number of load cycles**	**Percent of control (%)**	**Parameters**	**Loading form**	**Animal model**	**Reference**

1	40	−10	BFR	Four point bending	Rat tibia	Cullen et al.^248^
	120	25				
	400	39				
2	36	9	Ct.Ar	Axial compression	Mice tibia	Yang et al.^40^
	216	13				
	1200	15				
3	4	3	Bone mass	Trained to jump	Rat tibia	Umemura et al.^46^
	36	33				
	360	34				
	1800	43				
4	5	9.6	Bone mineral content	Isolated ulna	Rooster ulna	Rubin et al.^55^
	10	11.15				
	20	14.23				
	40	17.3				
	100	22.7				

## Appendix

### **2. Regression analysis methods**

Curve estimation regression analyses were conducted to assess the correlations between mechanical loading parameters (e.g., strain magnitude, loading frequency, strain rate, and number of load cycles) and bone adaptive changes to loading (e.g., rMAR, rBFR/BS, Ct.Ar, ect.). Here, linear, logarithmic, exponential, and power regression models were tested and an optimal regression model was selected for each mechanical loading parameter when the greatest determination coefficient (R2) was achieved. The curve estimation regression analyses were performed with SPSS (SPSS 25.0, IBM Inc., Chicago, USA) and curve fitting was done with GraphPad Prism 6 (GraphPad, Inc., San Diego, CA, USA).
